# Enhancement of Glen Moy x Latham raspberry linkage map using GbS to further understand control of developmental processes leading to fruit ripening

**DOI:** 10.1186/s12863-018-0666-z

**Published:** 2018-08-15

**Authors:** Christine A. Hackett, Linda Milne, Kay Smith, Pete Hedley, Jenny Morris, Craig G. Simpson, Katharine Preedy, Julie Graham

**Affiliations:** 1 Biomathematics and Statistics Scotland, Invergowrie, Dundee, DD25DA Scotland; 2 The James Hutton Institute, Invergowrie, Dundee, DD25DA Scotland

**Keywords:** Raspberry, GbS, Linkage analysis, QTL mapping, Hidden Markov model, Fruit development, Ripening

## Abstract

**Background:**

The changing climate is altering timing of key fruit ripening processes and increasing the occurrence of fruit defects. To improve our understanding of the genetic control of raspberry fruit development an enhanced genetic linkage map was developed and used to examine ripening phenotypic data.

**Results:**

In this study we developed an enhanced genetic linkage map for the raspberry cvs. Glen Moy x Latham reference mapping population using genotyping by sequencing (GbS). Alignment to a newly sequenced draft reference genome of red raspberry, cultivar (cv.) Glen Moy, identified 8019 single nucleotide polymorphisms (SNPs). After stringent filtering to take account of read coverage over all the progeny individuals, association with a single chromosome, heterozygosity and marker regression mapping, 2348 high confidence SNPs were retained and integrated with an existing raspberry genetic map. The linkage map contained many more SNPs segregating in Latham than in Glen Moy. This caused difficulties in quantitative trait loci (QTL) mapping with standard software and a novel analysis based on a hidden Markov model was used to improve the mapping. QTL mapping using the newly generated dense genetic map not only corroborated previously identified genetic locations but also provided additional genetic elements controlling fruit ripening in raspberry.

**Conclusion:**

The high-density GbS map located the QTL peaks more precisely than in earlier studies, aligned the QTLs with Glen Moy genome scaffolds, narrowed the range of potential candidate genes to these regions that can be utilised in other populations or in gene expression studies to confirm their role and increased the repertoire of markers available to understand the genetic control of fruit ripening traits.

**Electronic supplementary material:**

The online version of this article (10.1186/s12863-018-0666-z) contains supplementary material, which is available to authorized users.

## Background

Unpredictable phenotypic variation in a range of developmental traits directly impacts yield across a range of crops [1–6]. In raspberry, this has been evident in increased occurrence of crumbly fruit [4], lack of evenness and variable timings of bud break [7], flowering and fruiting [8], variations in yield, modification of primocane (annual) and biennial behaviour (Jennings pers. comm.) and a reduction in maximum photosynthetic capacity [9, 10]. Predictable variations in flowering and fruiting time across seasons and between varieties allow for grower scheduling across the season, but recently growers are also experiencing unexpected problems in the timing of fruit maturity affecting commercial production. Wild populations, which already exhibit differences in developmental transitions across a spatial scale, are also affected [11–14]. Raspberry marker assisted breeding uses genetic linkage maps to develop markers and identify genes associated to important crop characteristics. Maps have been constructed for diploid red raspberry crosses and black raspberry crosses [15–18], a cross between red and black raspberry [19] and a cross between the closely related primocane-fruiting and thornless tetraploid blackberry (*Rubus* subgenus *Rubus* Watson) [20]. Available raspberry genetic maps show synteny with related species in *Rosaceae*. For example, six of the seven linkage groups in cv. Latham are associated with *Fragaria* linkage groups (FLG) FLGVII, FLGIII, FLGVI, FLGII, FLGV and FLG1 and nearly 90% of the sequences of red raspberry markers tested aligned to the recent black raspberry genome assembly [19, 21].

The genetic linkage map developed from a cross between red raspberry cultivars Glen Moy and Latham, followed by QTL mapping, has identified multiple markers for a broad range of important raspberry agronomical characteristics and has led to improved cultivars [4, 7, 15, 22–30]. QTL analysis for developmental stages from bud break through to ripe fruit has identified underlying genes of potential significance for a number of important fruit ripening related traits including fruit colour, anthocyanins, flavour volatiles and progression of fruit softening [7, 23, 24, 27–30]. Previous maps of this cross show higher number of heterozygous markers in Latham (the previous map had 301 heterozygous markers from Latham, 85 from Glen Moy and 53 segregating in both parents), which is an early cultivar released in 1914 with traits close to species material [8] compared to the highly bred cultivar Glen Moy. The number of useful markers derived from the Glen Moy and Latham population compared to other crop species is, therefore, comparatively low. To exploit the full potential of this mapping population and enhance selection efficiency there is a need to increase the proportion of SNPs segregating in Glen Moy and construct a high-quality linkage map.

Genotyping by Sequencing (GbS) specifically targets identification of sequence variants around restriction enzyme sites. While GbS does not cover the entire genome, it allows rapid, cost effective and broad coverage targeted sequencing of any organism. GbS has previously been used in red raspberry to develop a saturated linkage map from a Heritage x Tulameen mapping population utilising 7000 SNP markers spanning all seven raspberry linkage groups [17]. Here we have used GbS to substantially increase marker coverage and construct a high-quality linkage map between Glen Moy and Latham using GbS SNPs together with existing markers. To improve positioning of the GbS sequences and identification of variants we also sequenced and assembled a draft genome for raspberry cv. Glen Moy. We re-analysed previous raspberry ripening data from the same population to further understand the genetic control of the processes controlling raspberry fruit development. The enhanced genetic linkage map and QTLs along with Glen Moy genome scaffolds locate important genomic regions and underlying candidate genes involved in these processes.

## Methods

### Material generation and DNA extraction

The raspberry mapping population consists of 188 individuals derived from a cross between the European red raspberry cv. Glen Moy and the North American red raspberry cv. Latham [15]. These two raspberry varieties differ in a large number of traits including plant architectural traits, pest and disease resistances, particularly root rot resistance, seasonality and fruit quality traits making this a wide cross allowing mapping of a large number of QTL. Genomic DNA was extracted from the parents and progeny using the same method as described previously [13].

### Construction of Glen Moy draft genome assembly

A fragmented genomic DNA library was prepared from cv. Glen Moy using a TruSeq DNA polymerase chain reaction (PCR)-Free Library Preparation Kit (Illumina). DNA fragmentation was completed using the Covaris microTUBES in the S220 (Covaris) (settings 10% Duty Factor, 175 W Peak Incident Power, 200 cycles per burst and 1 to 3 cycles of 1 min time) to generate fragments in the range of 200–300 bp. DNA fragment size was verified using the Agilent Bioanalyzer High Sensitivity DNA Kit (Bioanalyzer 2100, Agilent). Libraries were constructed according to the manufacturer’s recommendations (Illumina), prior to final quality control checking on a Bioanalyzer. Sequencing was performed as two independent runs on a MiSeq (Illumina) using paired-end 2 × 300 bp v3 kits, as recommended (Illumina).

An additional mate-pair library was constructed to assist scaffolding of genomic contig sequences. A Nextera Mate Pair kit (Illumina) was used as recommended by the manufacturer from the same genomic DNAs used for the standard library preps (see above). Final libraries were QC’d using the Agilent Bioanalyzer High Sensitivity DNA Kit on a Bioanalyzer 2100 (Agilent), prior to being sequenced on a single MiSeq run using a paired-end 2 × 150 bp v2 kit.

Reads were trimmed to Q28 and minimum length 80 bp with Sickle, sliding-window, adaptive, quality-based trimming tool for FastQ files (Version 1.33) (https://github.com/najoshi/sickle). PE reads were joined using clc_overlap, creating a set of joined and unjoined PE reads. The mate-pair MiSeq run was also trimmed with Sickle to Q28 minimum length 80 bp and the orphaned reads were included in the assembly. The trimmed mate-pairs were adapter trimmed using NextClip (v 1.3.1, [31]). A total of 13.7 Gbp were assembled using clc_assembler from the CLCBio suite (v 4.10.86742, https://www.qiagenbioinformatics.com/products/clc-assembly-cell/), with a range of k-mer sizes. The assembly with the best N50 (20,628 bp) was scaffolded using the mate-pair data with SSPACE (v 2.3). Scaffolding pre-assembled contigs using SSPACE with default parameters [32]. This gave a draft genome for Glen Moy of 147,546 output scaffolds covering 361,105,105 bp of an estimated 280 Mb genome [33]. To assess genome completeness, we compared our scaffolds against plant near-universal single-copy orthologs using Benchmarking Universal Single-Copy Orthologs (BUSCO) (https://busco.ezlab.org/) with default parameters and the plant dataset provided from the BUSCO website. The results showed that over 90% of the 1440 BUSCO groups were complete in the scaffolds, which is an indication that the assembly is of good quality. The draft Glen Moy genome assembly is available from the authors on request. The Glen Moy scaffolds were used as a reference assembly for mapping GbS reads.

### GbS library construction and sequencing

GbS libraries were constructed in a similar manner to Poland et al. [34] and as described in Russell et al. [35] for blackcurrant. The same set of 48 barcoded adapters and common Y-adapter used for GbS in blackcurrant was used. Annealed barcoded adapters were normalised to 2 ng/μl and the Y-common adapter to 40 ng/μl. 200 ng of genomic DNA from both Glen Moy and Latham and 188 progeny from the cross were independently digested with *Pst*I and *Mse*I and the reaction terminated at 80 °C for 20 mins. Groups of 48 of the digested DNAs were each ligated to 4 ng of annealed barcoded adapter and 200 ng of annealed Y-common adapter, incubated at 22 °C for 2 h, and the ligation terminated at 65 °C for 20 min. A tenth of this reaction was removed from each ligation reaction, pooled, purified using QIAquick PCR Purification Kit (Qiagen) and eluted in 30 μl of dH_2_O. This process was repeated three further times for the remaining DNA samples to give 4 × 48 samples (184 progeny and 4 each of Glen Moy and Latham). After PCR enrichment using primers complementary to the barcode adapter and Y-common adapter, the library was size fractionated in the 200 bp–500 bp size range, and the quality and quantity of the library was measured using Nanodrop (Thermo Scientific) and Bioanalyzer 2100 (Agilent).

Single-end 100 bp sequencing from the *Pst*I sites was carried out on 4 lanes (48 samples each) on a Illumina HiSeq2000 as recommended. The GbS data from the raw FastQ files were deconvoluted into separate files for each sample using the barcoded adapter sequences. The parental lines were included in each Illumina run, and so their files from each run were concatenated into a single file for each parent. The GbS reads from the GAII protocol were prepared for quality trimming by converting the quality score offsets (Illumina v1.5) to the same offset as the HiSeq2000 runs (Illumina v1.9). A custom pipeline was used to quality trim (Q28), length trim (64 bp), and map each sample’s reads against the reference set using the BOWTIE mapper with one mismatch allowed per read, and in “unique best strata” mode where each read is mapped only once. All the Binary Alignment/Map (BAM) outputs for each sample were merged into a single BAM file. Quality trimming to Q28 and length 64 bp retained 90–99% reads, except for two samples which gave 84% and 86% read retention. After quality trimming the parental lines gave 9,083,886 reads for Latham and 15,633,118 reads for Glen Moy. Sequencing of the 188 progeny produced 375,772,511 reads in total representing approximately 2.4 Gbp.

### SNP discovery

Two strategies were employed for SNP discovery. The first method was to map the GbS tag sequences against the newly created Glen Moy reference genome assembly, and the second was to use the UNEAK pipeline from the TASSEL suite of software [36].

#### SNPs from reference mapping

The EMBOSS utility ‘restrict’ (v 6.6.0.0, EMBOSS: The European Molecular Biology Open Software Suite (2000) [37] was used to find all *Pst*I sites in the Glen Moy sequence scaffolds and sequence 64 bp both sides of each site were extracted to make a library of 124,058 reference tags to map the GbS reads. After converting the reference tags into a Bowtie binary, the quality trimmed GbS reads for each sample were mapped with Bowtie (v 0.12.9 [38]) to the reference tags, allowing a single mismatch and a single reported mapping. Mapping placed 79.4% of the Glen Moy reads on to the Glen Moy reference assembly and only 60.7% of Latham reads were mapped due to the stringency of the Bowtie mapping and the higher degree of sequence variants. The BAM files from the Bowtie mappings were then merged into a single BAM file and analysed with FreeBayes (v 0.9.14 [39]) to find SNPs.

#### De novo SNP discovery with the UNEAK pipeline

The UNEAK pipeline (part of the TASSEL v3.0 pipeline, https://tassel.bitbucket.io/TasselArchived.html, [36] was also used for de novo SNP discovery from the raw GbS data, using default parameters.

### Linkage analysis of the mapping population

An iterative approach was used for map estimation and checking (Additional file 1: Appendix A) and the steps are described in more detail below.

#### Preliminary filtering of the SNP data

The 8019 SNPs from the Glen Moy reference mapping were filtered to obtain a high-quality set for the parents and 184 offspring of the Latham x Glen Moy cross. Filtering steps were used based on criteria which gave an informative set of SNPs in a population of blackcurrant [35]:

(i) Retain only SNPs with an average of 10 or more reads per individual i.e. a total read count of at least 1840.
(ii) Retain only SNPs with quality score ≥ 10,000.
(iii) Remove SNPs with % heterozygosity ≥90%.

The allele read counts of the retained SNPs were used to construct the linkage maps.

#### Preliminary allocation to chromosomes

As there is a well-established linkage map of the seven chromosomes for this population [4], a preliminary allocation of the high-quality SNPs remaining after filtering was made according to their degree of association with this map. To estimate this, the proportion P_A_ of the major allele read counts out of the total read counts was calculated for each SNP. If the parental genotypes are AB x AA or AA x AB, with A being the major allele and B the minor allele, then the offspring are expected to be AB and AA in equal proportions, and the proportion of the major allele P_A_ should take values close to 0.5 or 1.0. If the parents are AB x AB, and A is the major allele, the offspring are expected to be AA, AB, BB in a 1:2:1 ratio and the P_A_ should take values close to 1.0, 0.5, 0.0 respectively.

One-way analysis of variance was then used to test the association of P_A_ for each SNP with each of the markers in the existing map, measuring the degree of association by the significance of the analysis of variance F-test and the % variance explained (R^2^), to find the map position with the greatest association (this is a parametric version of the Kruskal-Wallis test used by MapQTL [40], sometimes referred to as marker regression mapping). SNPs were tentatively allocated to a linkage group if they showed an association with R^2^ greater than 25% for just one group.

#### Genotype identification

For the SNPs showing an association with just one linkage group of the existing map, SNP genotypes were estimated from the allele read counts using the functional regression approach developed for blackcurrant by Russell et al. [35]. Briefly, this plots the major and minor allele read counts as (X,Y) co-ordinates on a scatter plot. There is generally a clear grouping of individuals, with some (the assumed heterozygotes) forming an inclined line while the assumed homozygotes lie close to the horizontal and/or vertical axes. The relationship between the major and minor allele counts was modelled for the assumed heterozygotes by fitting a functional regression model [41] to the square root of the counts data, to stabilise the variation and to take into account the random variation in both sets of counts. Each offspring was then classified as AA, BB or AB depending on whether it was closest to the horizontal, vertical or inclined line. For offspring with both major and minor allele counts less than or equal to one the genotype was designated as missing.

To assess the fit of the classification for each SNP, the proportion of the major allele for the parents and offspring was regressed on the genotype classification and R^2^, the percentage variance explained, was calculated. Classifications were assessed as unsuitable for linkage mapping if they were monomorphic, or if R^2^ was less than 50%, or if the classification was considered clearly incompatible with the parental genotypes. These analyses were performed using Genstat 17 (GenStat for Windows 17th Edition 2014. VSN International, Hemel Hempstead, UK. GenStat.co.uk).

#### Linkage analysis

The GbS SNPs that were suitable for linkage analysis were combined with the marker data on that chromosome in the existing map and analysed with JoinMap 4.1 [42] to estimate the recombination fractions and logarithm of the odds (LOD) scores between each pair of markers. The maximum likelihood ordering approach of JoinMap is prone to substantial map inflation if there are many genotyping errors in the data [43], and the full regression mapping using three mapping rounds is slow for large datasets. The linkage maps were therefore constructed using two approaches: regression mapping in JoinMap using only two mapping rounds, in order to place the most reliable markers, and using a multi-dimensional scaling (MDS) approach, as described by Preedy and Hackett [44]. This was developed for rapid ordering of larger numbers of markers. It displays a two- or three-dimensional configuration of the markers that optimises a stress criterion very similar to the weighted least squares criterion used by the regression mapping algorithm of JoinMap. Preedy and Hackett [44] compared configurations obtained by using different functions of the LOD score as weights and found that LOD^2^ weighting was best for their simulated tetraploid data; here we compared LOD and LOD^2^ weighting in two or three dimensions in a similar way. For each weighting, outlying points in the MDS configurations were removed, the MDS analysis was rerun and the configuration of the remaining points was mapped to obtain a linear order by fitting a principal curve through it. The stress criterion is not comparable between the different weightings, and so marker orderings are compared on the basis of their mean nearest-neighbour fit (NNfit). This is calculated as the sum of the absolute difference between the observed and estimated map distance between that marker and the nearest informative neighbours on either side – that is the nearest neighbours with a non-zero LOD score. (Neighbouring markers where different parents are heterozygous are uninformative about recombination).

#### Map checking using a hidden Markov model

Once the markers were ordered, the map, of *m* markers, was checked by examining the ordered genotype scores. Hackett et al. [45] describe how a hidden Markov model (HMM, [46]) can be used to reconstruct the chromosomal states underlying each offspring’s genotype scores, and a summary is given in Additional file 2: Appendix B. Here, the HMM was run for each offspring to identify the chromosome configuration most likely to give the observed genotypes. This gave an *m × o* matrix of recombination locations, where *o* is the number of offspring. From this matrix, we then calculated how the total number of recombinations across all offspring was affected by: (i) excluding each marker in turn; (ii) swapping each marker with the adjacent marker and; (iii) trying all other possible orderings of the surrounding triplet of markers. The third approach was motivated by the RECORD software [47], and assumes that a badly scored or misplaced marker will have an unusually high number of recombinations in its vicinity in the chromosomal configuration, and that the order can be improved by removing it or by a local swap in ordering. Orders can be compared using HMM_mean, equal to the mean over all offspring of the recombinations that can be removed by excluding that marker.

### QTL mapping

Data on raspberry developmental stages measured on the Glen Moy x Latham population in 2006 at two sites, an open field and a polytunnel [7], were re-analysed. The ripening data was collected as visual assessments from bud break to over-ripe fruit using a 1–7 scale (1 = bud break, 2 = open flowers, 3 = fruit set, 4 = green fruit, 5 = green/red fruit, 6 = red fruit and 7 = over-ripe fruit). The phenotypic observations were taken between the months of May and July. In the field trial there was an additional estimation of the proportion of fruit that had not ripened on the last scoring date, and in the protected trial there was an additional assessment of the % open flowers in mid-June. As detailed in Graham et al. [7], ripening profiles for each location were summarised using a principal co-ordinate analysis (PCO) of the plot scores, based on a similarity matrix using the city-block metric. Principal coordinates PCO1, PCO2, PCO4 and PCO5 had significant differences among the offspring genotypes (*p* < 0.001) and were used for QTL mapping. PCO1 was interpreted, based on its correlations with the individual scores, as an overall summary of ripening speed (with high values associated with a longer ripening speed), PCO2 as a comparison between later and earlier scores so that a high value indicates a slow development in May and a rapid ripening in late June and early July, and PCO4 and PCO5 being similar to PCO2 but comparing dates in June and July. Principal coordinates have the advantage of being uncorrelated with each other, but do not have an intuitive meaning and so the time (in days) to reach each of stages 2 to 6 was interpolated from the ripening scores. For stages 2–5 there were significant differences (*p* < 0.05) among the offspring genotypes, and these were also used for QTL mapping.

Initially QTL mapping on the GbS map was carried out using interval mapping in MapQTL 5 [40]. This used the default interval mapping parameters, calculating QTL genotype probabilities based on genotypes at up to five neighbouring markers, but the maximum number of iterations of the model-fitting process was set as its minimum number of 10 to avoid overfitting the model. However, the LOD profiles from MapQTL were unexpectedly irregular, given the high-density map, resulting in uncertainty in locating the peak LOD score. Two other QTL mapping approaches were then tried for mapping the ripening data. The first used the QTL interval mapping routines in Genstat. The probabilities of each QTL genotype were calculated using the routine QIBDPROBABILITIES, followed by an initial genome-wide scan with the procedure QSQTLSCAN to identify candidate locations, a second scan with the same procedure using the initial locations as cofactors to test for additional candidate locations or improve their estimated positions, and finally the procedure QSESTIMATE was used to estimate the QTL effects at the selected locations. The second fitted an HMM approach adapted from its similar use for QTL mapping in autotetraploid species [45]. Details of fitting HMM for estimating QTL genotype probabilities in a diploid cross are given in Additional file 2: Appendix B.

### QTL simulation study

A small simulation study was conducted to compare the behaviour of the three QTL mapping methods for a population of the type and size used in the current study. The first simulations used the map estimated for LG2, simulating complete marker data without missing values, segregation distortion or genotyping errors. This linkage group was chosen as having the highest proportion of markers from the Glen Moy parent, at 20%. A single QTL of the size observed for PCO4 was simulated on this linkage group. The second set of simulations differed from the first in using a linkage map from a GbS map of blackcurrant [35], in which there were similar numbers of markers segregating in each parent.

### Linking trait QTL to genome scaffolds and underlying genes

Once the QTL mapping was complete, Glen Moy genome scaffolds within a 2 cM interval on either side of the most significant marker were identified. These genome scaffolds were compared to the Arabidopsis genome using the TAIR Bulk Data Retrieval Tool to conduct a GO annotation search (https://www.arabidopsis.org/tools/bulk/go) to identify genes with a potential role in the fruit developmental process. Two non-related map regions were also examined to ensure developmentally and ripening related genes were not identified randomly.

## Results

### Glen Moy draft genome reference

To facilitate reference sequence-based assembly of GbS sequence tags, a draft genome sequence was established for the raspberry cultivar Glen Moy. Combined paired-end and mate-pair sequencing for maximum sequence coverage across the genome produced 14 Gbp, representing approximately 50 X coverage of the predicted 275 Mbp genome. A total of 147,546 scaffolds were constructed covering 360 Mbp with the largest scaffold 750 Kbp. To assess genome completeness we ran BUSCO (https://academic.oup.com/bioinformatics/article/31/19/3210/211866) to compare our scaffolds against a plant dataset of near-universal single-copy orthologs. Of 1440 orthologs searched, 90.5% were complete, 3.7% were fragmented and 5.8% were absent. Overall this draft genome represents good coverage of the red raspberry genome and its gene space and was used as a reference genome to identify SNPs.

### Genotyping by sequencing

To substantially increase marker coverage, a high-quality linkage map between Glen Moy and Latham was constructed using the GbS identified SNPs together with existing markers. After removing adapters and quality score trimming, sequencing of the parental lines and 184 individuals from the full sib population resulted in over 400 million reads consisting of 9,083,886 reads for Latham and 15,633,118 reads for Glen Moy. The average number of reads for each progeny clone was 2,042,242, ranging from a few hundred to over 8.5 million reads. Of the population, 33 progeny clones had fewer than 100,000 reads and were removed from the analysis and 27 had between 100,000 and 1 million reads. The remaining 124 progeny clones had over 1 million reads each. From the reference-mapped SNPs, after basic filtering, FreeBayes found 8019 potential SNP sites and 5856 of these had a read depth of at least 1000. From the UNEAK pipeline a total of 20,742 SNPs were discovered and of these 7919 had a read depth of at least 1000. The SNP-containing tags from UNEAK were compared to the reference-mapped SNPs to verify the overlap between the two sets of SNPs, with a subset of the matches being checked by hand using Tablet [48]. 5935 of the UNEAK SNPS were detected within the reference SNP set, and 4371 of these have at least 1000 reads. Also 5823 UNEAK SNPs matched a reference *Pst*I site, but no SNP was called by the reference assembly method.

### Selection of segregating SNPs

The 8019 SNPs identified against the reference genome were further filtered to obtain a high-quality set segregating in this population. Filtering to retain only SNPs with an average of 10 or more reads per individual reduced the set to 4992 SNPs. Filtering to retain only SNPs with a Freebayes quality score ≥ 10,000 reduced the set to 4954 SNPs and removing SNPs that were scored as heterozygous in more than 90% of the population reduced the final selected set to 4437 SNPs. The major allele proportion, *P*_*A*_, was calculated for each of the retained SNPs and mapped onto the existing linkage map using marker regression mapping, to identify the most closely linked marker, its map position and the percentage of variance (R^2^) in *P*_*A*_ that was explained by this marker. Inspection of the overall distribution of R^2^ showed a bimodal distribution with peaks close to 5% and 75% and showed that a threshold of R^2^ ≥ 25% would be a reasonable choice for allocating SNPs. Of the 4437 SNPs, 336 had R^2^ < 25% and were not allocated to any group, 41 showed associations with more than one linkage group and the remaining 4060 showed an association with just one chromosome (Table 1).

**Table 1:** Association of GbS SNPs to the existing raspberry linkage map, showing distribution by parental classes

Chromosome	No. SNPs allocated by ANOVA (R^2^ > 25%)	No. ABxAA	No. AAxAB	No. ABxAB	No. AOxAB or ABxAO	Not called
LG1	439	275	40	5	39	80
LG2	653	361	102	7	58	125
LG3	905	559	44	9	101	192
LG4	618	311	69	28	65	145
LG5	661	327	58	16	95	165
LG6	451	355	17	0	15	64
LG7	333	270	0	0	15	48
More than one chromosome	41					
Unallocated	336					
Total	4437	2458	330	65	388	819

AB x AA denotes that markers that are heterozygous in Latham only, AA x AB denotes markers that are heterozygous in Moy only, AB x AB denotes markers that are heterozygous in both parents and AOxAB or ABxAO denotes markers where the segregation ratio is consistent with a null allele (O) in one parent

### Genotype identification

For the 4060 SNPs with an association to one chromosome, genotypes were called from the allele read counts using a functional regression approach [35]. The genotype proportions for each genotype class were inspected and SNPs with the following three categories were tentatively identified: 1. Heterozygous in Latham, homozygous in Glen Moy, offspring showing two genotype classes, coded as JoinMap type AB x AA (2458 SNPs); 2. Heterozygous in Glen Moy, homozygous in Latham, offspring showing two genotype classes, coded as JoinMap type AA x AB (330 SNPs); 3. Heterozygous in both Latham and Glen Moy, offspring showing three genotype classes, coded as JoinMap type AB x AB (65 SNPs). The majority of the chosen SNPs were heterozygous in the Latham parent only. In addition to these expected types, there were 388 SNPs that showed a pattern of being heterozygous (AB) in one parent, apparently homozygous (AA) in the other but with three offspring genotype classes AA, AB, BB, segregating in an approximate 2:1:1 ratio. This segregation pattern was also found by Russell et al. [35] and is consistent with a null allele (O) for the ‘homozygous’ parent, i.e. the genotypes of the given locus in the parents are actually AB and AO, with offspring genotypes AA: AO: AB: BO in an expected 1:1:1:1 ratio and therefore showing phenotypes A only, AB and B only in a 2:1:1 ratio. As standard software such as JoinMap does not handle AB x AO markers, these were recoded as two separate alleles, with the B allele having an expected 1:1 segregation ratio and the A allele having a dominant 3:1 segregation, as AA and AO cannot be distinguished. The A allele is not very informative in this case, but due to the low numbers of 1:1 markers from Glen Moy we used all information about segregation in Glen Moy. The initial ‘s’ in the SNP name was replaced by ‘n1’ or ‘n3’ for the B and A alleles respectively to identify them during the linkage analysis. Table 1 shows the number of SNPs of each type, for each chromosome separately. The remaining 819 SNPs did not fall into any of these categories and were not carried through to the linkage analysis in JoinMap.

### Linkage analysis – First round

The markers from the four genotype classes described above were combined with the existing mapped markers on each chromosome. The resulting markers were read into JoinMap, for each chromosome separately (Table 2). Duplicate markers, markers with more than 40 missing values and markers with JoinMap’s highest level of segregation distortion (*p* < 0.0001) were excluded. JoinMap was used to calculate the recombination fractions and LOD scores between all pairs of remaining markers within each chromosome. This pairwise data was ordered using multi-dimensional scaling (MDS) [44]. Outlying SNPs in the MDS configuration were removed and a principal curve was fitted through the remaining markers to give a preliminary GbS map. Table 2 shows the distribution of markers of each type on this map.

**Table 2:** Counts of markers used for first round of mapping, including markers from map in [4]

Chromosome	No. markers from Graham et al. (2015)	Total No. of markers in JM input file	No. carried to MDS analysis	Mapped markers	Latham (ABxAA)	Moy (AAxAB)	Both (ABxAB +ABxCD)	Null (AOxAB or ABxAO)
LG1	48	446	270	265	206	33	7 + 3	16
LG2	86	672	407	372	249	83	7 + 4	29
LG3	98	912	507	486	399	43	8 + 4	32
LG4	50	588	327	320	207	61	20 + 5	27
LG5	65	656	380	357	240	45	14 + 6	52
LG6	65	467	289	255	253	0	1	1
LG7	27	327	192	176	171	0	0	5

*JM* JoinMap

#### Addition of further markers with null alleles

In addition to the SNP pattern believed to be derived from AB x AO or AO x AB parental genotypes, a further pattern was observed during the genotype calling in which the three offspring phenotypes AA, AB and BB segregated with an approximate 1:1:1 ratio, and the parents had the phenotypes as different homozygotes, which should lead to no segregation in the offspring. To study these, 28 SNPs following this pattern and showing an association by marker regression with LG5 were selected from the 819 SNPs that were initially not classified. The read counts for each allele (major or minor) were analysed separately to see whether they were related to the additive effect of Latham, the additive effect of Glen Moy, or the dominance effect. In all but two of the 28 SNPs, the minor allele was associated with the additive effect from one parent (usually Glen Moy) and the major allele was associated with the additive effect of the other parent. We hypothesise that this is a different configuration of null alleles, and that the parents for the said loci are AO x BO, which is compatible with their observed phenotypes. This scenario would be expected to give equal numbers of offspring genotypes AO, AB, BO and OO. However, the OO would have been called as missing values, leaving approximately equal proportions of the other phenotypes as observed. An examination of the distribution of missing values for each SNP confirmed that there were higher numbers of missing values for these OO markers.

These SNPs can be included in the linkage analysis, but to code them for JoinMap analysis requires a tentative identification of which offspring have missing values arising from OO genotypes and which are missing due to other biological and/or technical reasons. Inspection of the total number of missing values for each offspring showed a bimodal distribution, with most offspring having fewer than 10% missing values, but some having a much higher proportion. For offspring with fewer than 10% missing values in general, missing values among these null markers were assumed to be OO genotypes, while for other offspring these values were kept as missing. These AO x BO null SNPs were then recoded as JoinMap’s AB x CD marker type. The initial ‘s’ in the SNP name was replaced by ‘n4’ to identify them during the linkage analysis. The counts of these markers are shown in Table 3.

**Table 3:** Counts of markers used for the second round of the linkage analysis

Chromosome	Possible AOxBO nulls	Total No. of markers in JM input file	No. carried to MDS analysis	Mapped markers	Latham (ABxAA)	Moy (AAxAB)	Both (ABxAB +ABxCD)	Null (AOxAB or ABxAO)	Null (AOxBO)	Length (cM)	HMM_mean for MDS order	HMM_mean after swaps
LG1	25	471	331	295	223	33	7 + 1	19	12	114.1	1.48	1.25
LG2	65	737	494	456	286	90	10 + 4	34	32	107.1	1.65	1.42
LG3	21	933	603	557	447	45	9 + 4	39	13	128.2	1.77	1.67
LG4^a^	79	667	448	435	253	70	25 + 5	39	43	91.5	1.29	1.25
LG5	83	739	509	486	287	66	18 + 8	63	44	85.4	1.42	1.27
LG6	12	514	369	315	284	15	1 + 1	9	5	88.6	1.95	1.52
LG7	16	396	252	243	216	11	0	10	6	100.2	1.93	1.55

HMM_mean denotes the mean number of recombinations inferred using a HMM

^a^ The LG4 map is fitted using LOD^2^ weighting and 2d MDS, which fitted better than LOD^2^ weighting and 3d MDS

#### Linkage analysis – Second round

The JoinMap analysis was rerun on the extended set of markers, after relaxing the filtering on missing values so that only markers with 45 or more missing values were excluded. After calculation of the recombination fractions and LOD scores, two rounds of the JoinMap regression mapping algorithm were run to obtain a map of the best-fitting markers for each linkage group. Each group was also analysed using MDS to obtain an ordering using LOD and LOD^2^ weighting and inspecting configurations in two and three dimensions. For six of the seven linkage groups, using a three-dimensional configuration and LOD^2^ weighting gave the smallest value of the NNfit measure, while for linkage group LG4 the two-dimensional configuration and LOD^2^ weighting was slightly better.

For LG7 the map was least informative about the Glen Moy parent, with two AO x BO SNPs lying 14 cM apart being the only mapped markers segregating in this parent (although there were some AB x AO SNPs clustering with this group but unplaced on the MDS map). There were 366 SNPs initially unallocated to any linkage group (see Table 1) and these were further tested to see whether any could be Glen Moy (AA x AB) markers belonging here. Sixty-four of these were compatible with a 1:1 segregation ratio and 11 were placed on this map by MDS, along with an additional four ‘AO x BO’ markers. These have the initial letter ‘s’ replaced by ‘m’ on the maps to identify them.

**Fig. 1:**
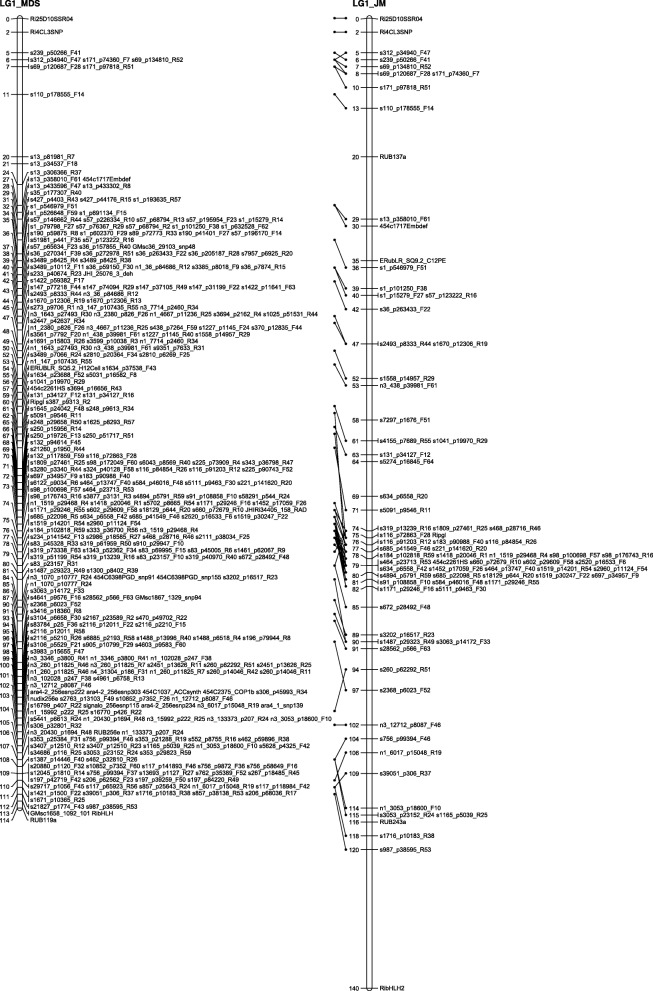
GbS linkage map of linkage group 1 of Glen Moy × Latham. Maps are ordered by MDS or by two rounds of JoinMap (JM)

**Fig. 2:**
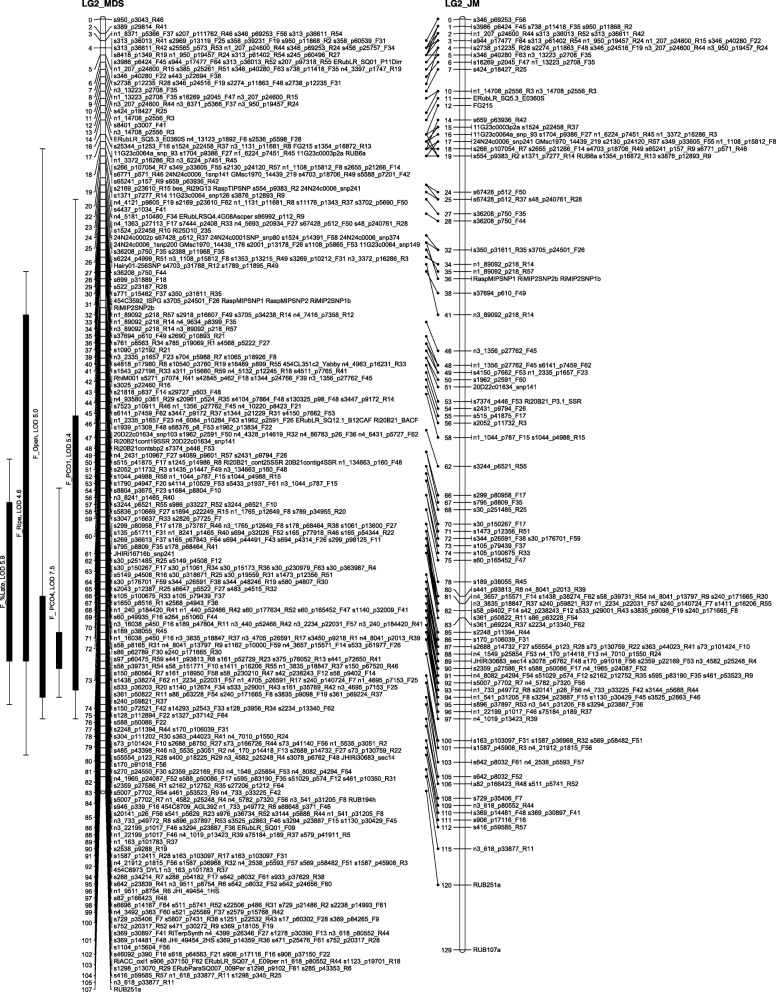
GbS linkage map of linkage group 2 of Glen Moy × Latham. Maps are ordered by MDS or by two rounds of JoinMap (JM). Black bars and whiskers (to the left of the chromosome) show one- and two-lod support intervals for QTL locations for developmental traits in 2006. F before a trait name indicates the field trial, P indicates the protected trial

**Fig. 3:**
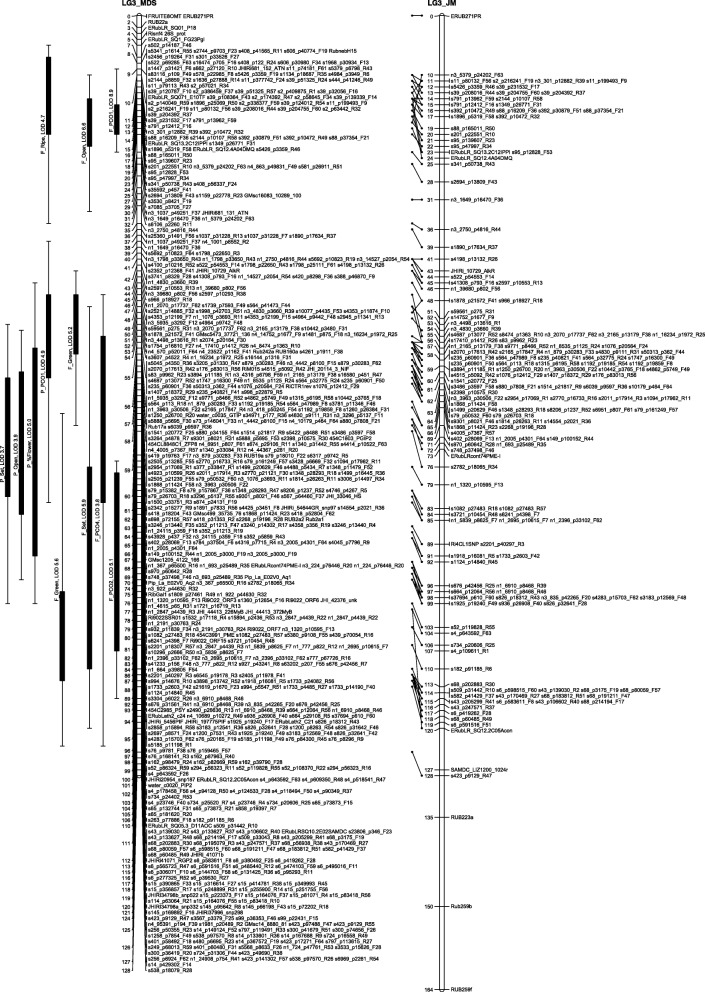
GbS linkage map of linkage group 3 of Glen Moy × Latham. Maps are ordered by MDS or by two rounds of JoinMap (JM). Black bars and whiskers (to the left of the chromosome) show one- and two-lod support intervals for QTL locations for developmental traits in 2006. F before a trait name indicates the field trial, P indicates the protected trial

**Fig. 4:**
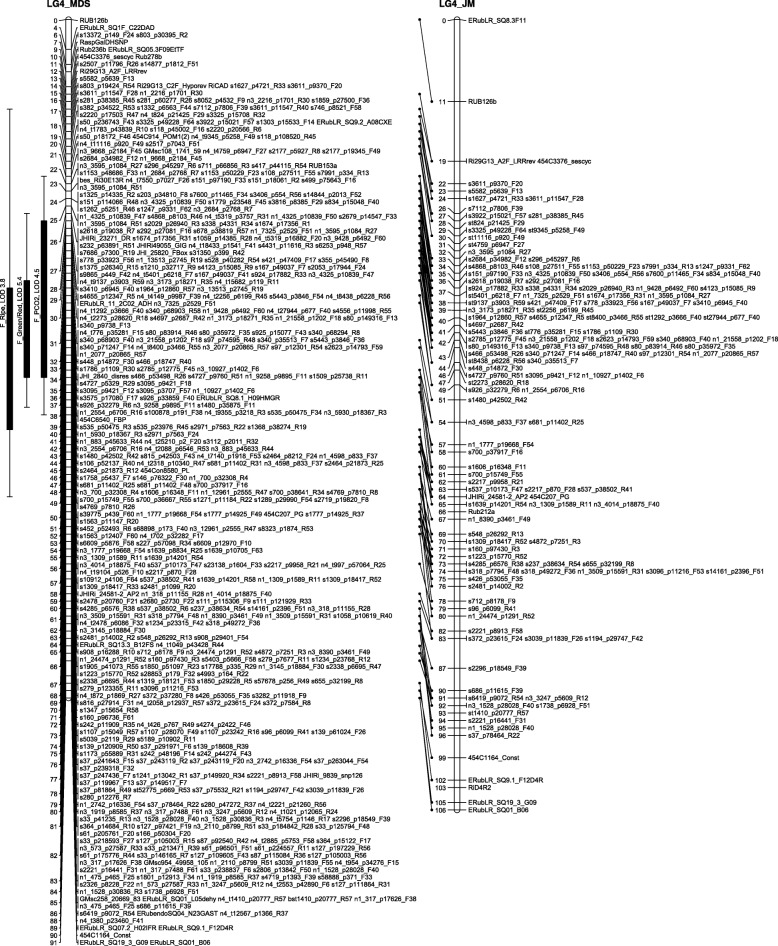
GbS linkage map of linkage group 4 of Glen Moy × Latham. Maps are ordered by MDS or by two rounds of JoinMap (JM). Black bars and whiskers (to the left of the chromosome) show one- and two-lod support intervals for QTL locations for developmental traits in 2006. F before a trait name indicates the field trial, P indicates the protected trial

**Fig. 5:**
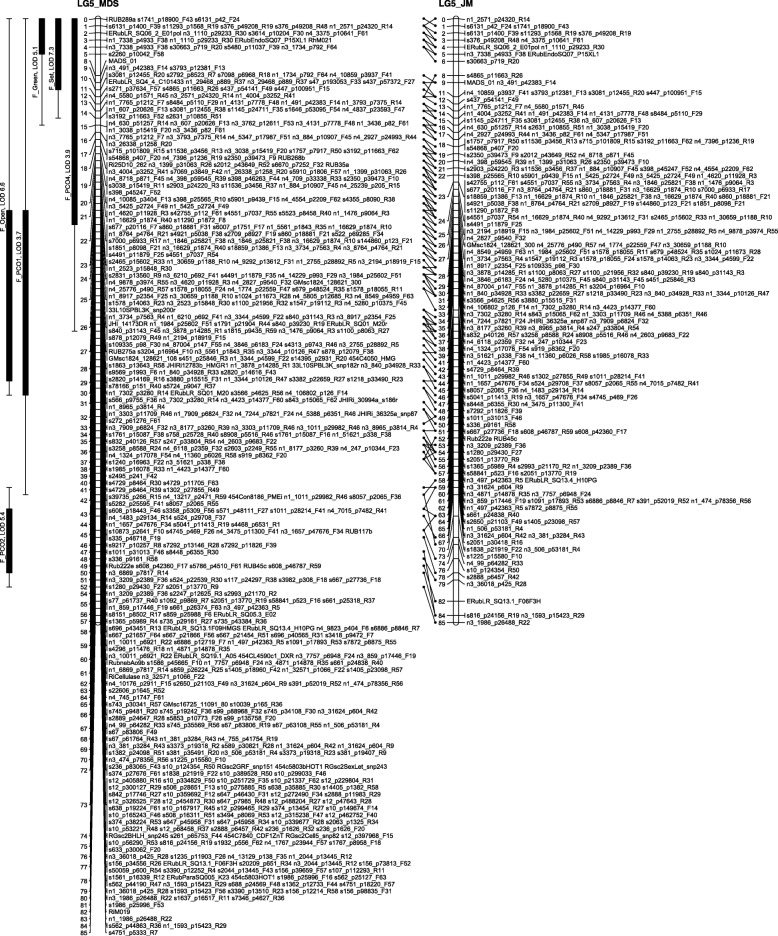
GbS linkage map of linkage group 5 of Glen Moy × Latham. Maps are ordered by MDS or by two rounds of JoinMap (JM). Black bars and whiskers (to the left of the chromosome) show one- and two-lod support intervals for QTL locations for developmental traits in 2006. F before a trait name indicates the field trial, P indicates the protected trial

**Fig. 6:**
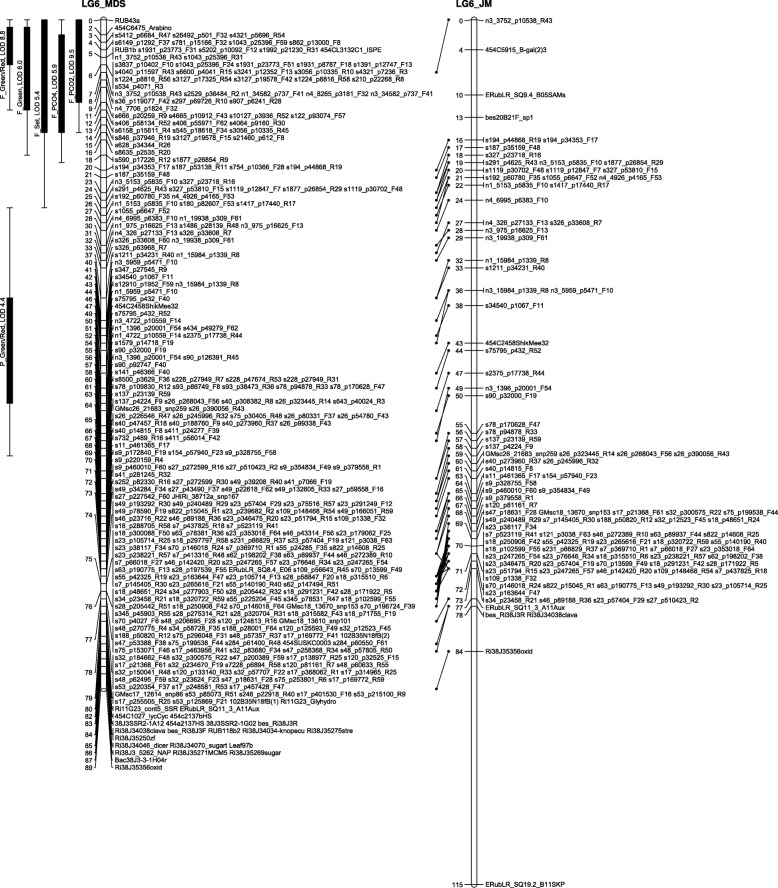
GbS linkage map of linkage group 6 of Glen Moy × Latham. . Maps are ordered by MDS or by two rounds of JoinMap (JM). Black bars and whiskers (to the left of the chromosome) show one- and two-lod support intervals for QTL locations for developmental traits in 2006. F before a trait name indicates the field trial, P indicates the protected trial

**Fig. 7:**
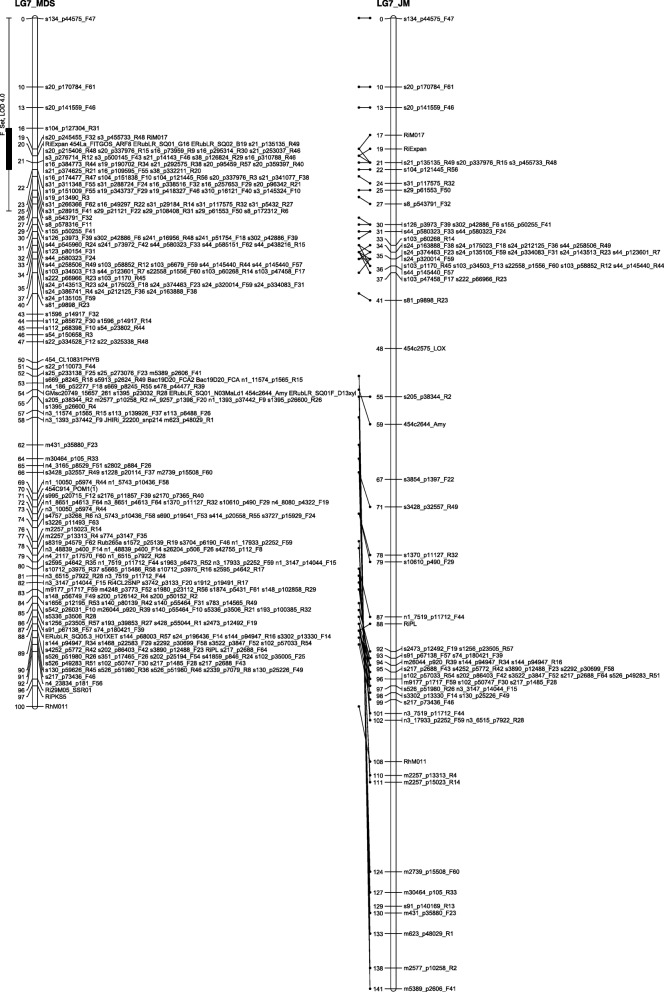
GbS linkage map of linkage group 7 of Glen Moy × Latham. Maps are ordered by MDS or by two rounds of JoinMap (JM). Black bars and whiskers (to the left of the chromosome) show one- and two-lod support intervals for QTL locations for developmental traits in 2006. F before a trait name indicates the field trial, P indicates the protected trial

The number of recombinations in the best map for each linkage group was investigated using HMM. No markers were identified as having an unusually high number of double recombinations, but a small number of local swaps were identified that reduced the overall proportion of recombinations. The markers with null alleles did not show any pattern of excessive recombinations with their neighbours. Comparison of the MDS maps (after swaps from the HMM) with the maps from the second round of JoinMap showed good agreement with only local rearrangements (Figs. 1, 2, 3, 4, 5, 6 and 7). The exception was LG7, where the additional Glen Moy markers (with labels starting with ‘m’) were placed by JoinMap at the end of the linkage group rather than distributed through it (Fig. 7). Table 3 shows summary statistics on the final maps, the breakdown of markers into categories and the improvements in the count of recombinations. The final map has 2787 markers, 2348 more than the map of [4].

### QTL simulation study

A small QTL study focused on understanding why the QTL profiles were unusually irregular using the above maps. Additional file 3: Fig. S1 gives profiles for mapping a simulated trait (with a single QTL at 67 cM) on problem-free markers simulated using the map of raspberry LG2, which has 62% of markers from the Latham parent only, 20% from Glen Moy only and 18% other types. Even with complete marker data, without genotyping errors or distorted segregation, the LOD profile from MapQTL was very uneven (Additional file 3: Fig. S1a). The profile from Genstat (Additional file 3: Fig. S1b) (using –log_10_(p) as the test statistic) is smoother, and that for the HMM (Additional file 3: Fig. S1c) is smoother still. A corresponding simulation from a blackcurrant map with 28% of markers from the first parent, 28% from the second parent and 44% of other types was carried out. The QTL in this case was simulated at 29 cM, with the same parameters as before and in this case the profiles were more similar for the different methods (Additional file 3: Figs. S1d, e and f). The marked dips in the QTL profile in Additional file 3: Fig. S1a at positions 60 cM and 72 cM occurred at positions where the genotypic information content for Glen Moy was close to zero. This study suggests that MapQTL’s approach for calculating genotypic probabilities has difficulty in the presence of unbalanced data from the two parents, as there may be little or no information about one parent’s genotype from the neighbours. This was marginally improved by increasing the MapQTL option *maximum number of neighbouring markers used* in the calculation of genotype probabilities, but the speed of mapping was substantially reduced: mapping four traits on this simulated chromosome with the default of five neighbouring markers took eight seconds while doubling this to ten neighbours increased the analysis time to 101 s.

### QTL analysis of experimental data

**Fig. 8:**
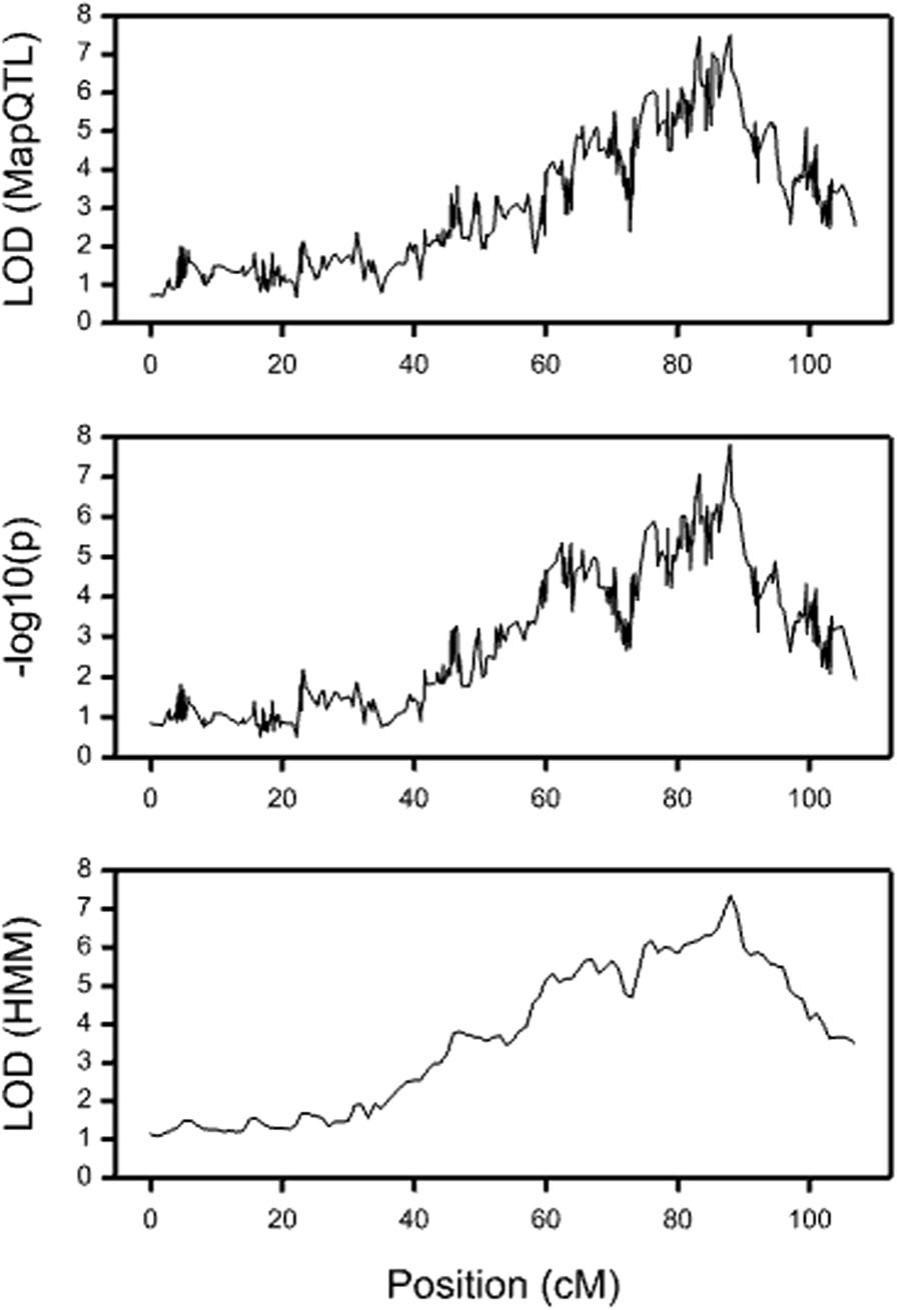
Likelihood profiles for the QTL for PCO4 on LG2.. Profiles are from MapQTL (top panel), Genstat (middle panel) and HMM (bottom panel)

Profiles obtained from linkage mapping of PCO4 from the ripening field trial data on LG2 using (a) MapQTL, (b) Genstat, (c) a HMM, for interval mapping indicate a significant QTL on this linkage group. The marker data differs from the simulated data above in having some marker data missing, but there were no regions of segregation distortion. MapQTL and Genstat show an irregular profile as found in the simulations (Fig. 8a and b). However, the use of a HMM continues to produce a smoother profile and a clearer peak location in the presence of missing marker data (Fig. 8c). The QTL analysis therefore used an HMM-based approach in the QTL interval mapping to identify the peak locations. A permutation test based on 200 permutations estimated that the genome-wide threshold for a significance level of 0.05 should be LOD = 3.7. Figs. 2, 3, 4, 5, 6 and 7 show one- and two-LOD support intervals for the QTL locations and Table 4 shows genotype means, LOD scores, % variance explained and closest SNP.

**Table 4:** QTLs detected using a HMM

Env	LG	Trait	Pos. (cM)	Mean_AC	Mean_AD	Mean_BC	Mean_BD	%var	LOD	se_AC	se_AD	se_BC	se_BD	Nearest SNP	Detected previously	Key parent
Field	2	Red	57	46.02	46.31	46.59	47.09	9.10	4.57	0.163	0.156	0.177	0.180	s986_p33227_R52	No	Both
Field	2	PCO1	63	−0.10	0.03	−0.04	0.10	10.27	5.42	0.029	0.029	0.032	0.032	s1650_p8516_R1	Yes	Both
Field	2	Open	85	13.36	15.03	13.69	15.16	9.99	5.03	0.309	0.336	0.335	0.338	s976_p36734_R52	No	Moy
Field	2	%late	86	0.06	0.09	0.27	0.28	11.58	5.91	0.036	0.039	0.038	0.039	s3294_p23887_F36	Yes	Latham
Field	2	PCO4	88	−0.01	−0.05	0.03	0.03	15.22	7.46	0.010	0.012	0.011	0.011	s22199_p1017_F46	Yes	Latham
Field	3	Red	8	46.17	45.97	46.81	46.89	9.64	4.70	0.161	0.174	0.167	0.169	s2456_p19264_F31	No	Latham
Poly	3	Red	9	50.68	51.00	51.73	51.77	14.70	5.25	0.171	0.210	0.197	0.196	s2456_p19264_F31	No	Latham
Field	3	PCO1	16	−0.11	−0.08	0.05	0.12	17.07	8.91	0.027	0.032	0.029	0.029	s88_p165011_R50	Yes	Latham
Field	3	Open	17	13.22	13.64	14.76	15.46	12.95	6.64	0.306	0.347	0.319	0.325	s88_p165011_R50	Yes	Latham
Field	3	Green	49	25.76	25.89	26.46	26.27	10.20	5.17	0.108	0.131	0.118	0.127	s59561_p275_R31	Yes	Latham
Poly	3	PCO1	56	−0.06	−0.09	0.06	0.08	12.06	4.95	0.025	0.033	0.026	0.036	s4830_p9111_R31	Yes	Latham
Poly	3	Open	62	21.10	20.03	22.55	22.42	8.68	3.91	0.368	0.554	0.427	0.524	s3246_p13440_F35	Yes	Latham
Poly	3	%flower	66	49.80	62.33	41.67	41.96	11.96	5.02	2.371	3.681	2.790	3.619	s2005_p3000_F19	Yes	Both
Field	3	PCO4	76	0.02	0.01	−0.04	−0.01	6.99	3.75	0.010	0.013	0.011	0.013	s4615_p65_R31	No	Latham
Poly	3	Set	76	24.96	25.17	26.88	25.96	8.51	3.73	0.314	0.451	0.376	0.462	s4615_p65_R31	Yes	Latham
Field	3	PCO2	94	0.06	0.00	−0.05	−0.05	9.98	5.10	0.017	0.019	0.018	0.022	s37694_p610_F60	Yes	Latham
Field	3	Set	102	21.33	22.15	22.72	22.92	11.17	5.96	0.215	0.252	0.251	0.293	s4_p124533_F28	Yes	Both
Field	3	Green	105	25.75	25.95	26.40	26.49	11.21	5.66	0.102	0.119	0.124	0.143	s65_p181620_R20	Yes	Both
Field	4	PCO2	35	0.01	0.02	−0.08	0.02	8.80	4.50	0.019	0.017	0.020	0.020	s2217_p870_F28	No	Both
Field	4	Green/Red	39	41.31	41.56	43.88	40.40	10.28	5.41	0.526	0.475	0.508	0.550	s702_p32282_F17	No	Both
Field	4	Red	42	46.52	46.45	46.94	45.90	7.49	3.81	0.172	0.155	0.172	0.181	s39775_p439_F60	No	Both
Field	5	Set	1	21.40	21.62	22.43	23.20	14.96	7.34	0.232	0.249	0.258	0.238	s376_p49208_R48	Yes	Both
Field	5	Green	1	25.70	25.95	26.27	26.45	10.15	5.08	0.114	0.123	0.128	0.118	s376_p49208_R48	Yes	Both
Field	5	PCO4	17	0.03	0.02	−0.03	−0.01	7.69	3.95	0.011	0.012	0.012	0.011	s54868_p407_F20	Yes	Latham
Field	5	PCO1	40	−0.07	−0.06	0.00	0.09	6.99	3.74	0.031	0.031	0.033	0.029	s608_p18443_F46	Yes	Both
Field	5	Open	40	13.46	13.57	14.23	15.64	13.08	6.62	0.318	0.328	0.346	0.307	s608_p18443_F46	Yes	Both
Field	5	PCO2	72	−0.05	−0.03	−0.01	0.08	10.87	5.42	0.020	0.017	0.018	0.020	s1838_p21919_F22	No	Both
Field	6	PCO2	3	0.06	0.07	−0.08	−0.04	19.26	9.48	0.019	0.019	0.018	0.016	s4321_p5696_R54	Yes	Latham
Field	6	Green/Red	3	40.48	39.57	43.35	43.14	17.75	8.76	0.521	0.531	0.492	0.436	s4321_p5696_R54	Yes	Latham
Field	6	PCO4	5	0.01	0.05	−0.04	−0.01	11.72	5.89	0.012	0.012	0.011	0.010	s5202_p10092_F12	Yes	Latham
Field	6	Set	9	22.02	21.11	22.85	22.44	10.47	5.44	0.263	0.268	0.251	0.225	s666_p20259_R9	No	Latham
Field	6	Green	9	26.06	25.53	26.38	26.24	11.61	6.04	0.126	0.128	0.120	0.107	s666_p20259_R9	No	Latham
Poly	6	Green/Red	46	47.03	47.46	49.35	49.05	11.69	4.43	0.452	0.430	0.416	0.455	s75795_p432_F40	No	Latham
Field	7	Set	20	21.65	21.45	22.66	22.62	7.70	4.00	0.271	0.267	0.240	0.240	s21_p135135_R49	No	Latham

*Env* environment, *LG* linkage group, Pos is the position of the maximum LOD in cM, Mean_AC etc. are the means of the four offspring genotype classes AC, AD, BC, BD (assuming a QTL model of Latham having genotype AB and Moy having genotype CD), se_AC etc. are the corresponding standard errors, %var. is the % trait variance explained by this QTL, ‘Detected previously’ indicates whether it was reported by Graham et al. (2009b) and ‘Key parent’ indicates whether one or both parents’ alleles are significant, based on pairwise t-tests of the estimated genotype means

### Fruit ripening QTL

The raspberry developmental stages data from [7] were re-analysed using the newly created high-density GbS-based genetic map (Glen Moy x Latham) with the aim of locating QTL peaks more precisely and identifying candidate genes. The description of phenotypic data, including other relevant details, are provided in the *Materials and Methods* section. Analysis of fruit ripening in the field, or under sheltered polytunnels, identified multiple different QTL for developing fruit at different ripening stages and whether they were grown in the open or protected environment (Summarised in Table 5; Additional file 4: Table S1). In total, 34 QTL were identified, the majority of which (28) were from the field data. On LG2, large QTLs (with LOD > 5) were confirmed for PCO4 (88 cM), late fruiting (86 cM) (both Latham effects) and PCO1 (63 cM), all from the field scorings. The significance of the QTL for PCO1 increased particularly, from a LOD 2.9 to 5.4, with significant effects from both parents. New QTLs were also identified on LG 2, for red fruit in the field (57 cM, with effects from both parents) and for open flowers (85 cM). The latter QTL is close to those for PCO4 and late fruiting, but this one shows a different effect (i.e. mainly Glen Moy, when others are mainly Latham). As in the previous analysis, LG3 had most QTLs. On LG3 at 17 cM there was a highly significant QTL (LOD 8.9) associated with rate of ripening in the field and this region was also associated with days to open flowers. PCO2, days to fruit set and days to green fruit in the field had QTLs at the bottom end of this linkage group viz. LG3 94 cM, 102 cM and 105 cM with LODs of 5.1, 6.0 and 5.7, respectively. New QTL on LG 3 were located at 8–9 cM for time to red fruit in the field and polytunnel and at 76 cM for PCO4 in field. All of the LG3 QTLs were mainly effects of the allele from Latham, apart from % flower in the polytunnel where there were significant effects from both parents. On LG5 the largest QTL was for the days to fruit set, with LOD 7.3 at LG5 1 cM and close to the previously identified marker RiMADS-01 positioned at 8 cM on this map. The QTL at 72 cM on LG 5 for PCO2 in the field is new. LG6 showed the largest QTL for the PCO2 in the field, with LOD 9.5 at LG6 3 cM. Other QTLs for PCO4 (LOD 5.9), days to set fruit (LOD 5.4), days to green fruit (LOD 6.0) and days to green/red fruit (LOD 8.8), were also found nearby. All were principally effects of the Latham allele. The QTLs for the earlier ripening stages fruit set and green fruit were not found in the previous analysis, and neither was the QTL for days to green/red fruit in the polytunnel at 46 cM. Two linkage groups, LG 4 and LG 7, had no QTL detected in the previous study but they were detected here (Table 4).

**Table 5:** Summary of traits linked to genes with roles in flowering and fruit ripening (full data available in Additional file 4: Table S1)

Trait	QTL	Genes identified with GO annotation: Biological processes
Days to open flowers	LG2 85 cM	carpel, flower, stamen embryo development
	LG3 17 cM	flowering, photoperiodism, flower, embryo development
	LG3 62 cM	embryogenesis, post-embryonic development, flower development
	LG5 40 cM	photoperiodism, embryo development
%Open flowers	LG3 66 cM	embryo development, post-embryonic development, pollen tube growth and recognition
Days to Fruit set	LG3 76 cM	negative regulation of flowering and floral meristem cell accumulation, cell wall organisation and signalling processes
	LG3 102 cM	embryo development, embryogenesis, pollen development, pollen recognition
	LG5 1 cM	embryo development, pollen development and cell wall development
	LG6 9 cM	embryo development and cell division
	LG7 20 cM	fertility, embryo development, flowering, pollen development and inflorescence development
Days to green fruit	LG3 49 cM	plant growth, signalling and seed coat development
	LG3 105 cM	embryo, endosperm and pollen development, pollen recognition, transport and signalling
	LG5 1 cM	regulation of flowering period, embryo development, pollen development and cell wall development
	LG6 9 cM	embryo development, cell division and regulation
Days to green/red fruit	LG4 39 cM	embryo development ending in seed dormancy, seed maturation and cell wall modification
	LG6 3 cM	cell wall organisation
	LG6 46 cM	fruit embryo development ending in seed dormancy
Days to red fruit	LG2 57 cM	post-embryonic development, embryo development ending in seed dormancy and cell growth
	LG3 8 cM & 9 cM	embryo development, floral organ morphogenesis, seed development, cell expansion, cell wall organisation negative regulation of flowering
	LG4 42 cM	pectin lyase, cell wall organisation, embryo seed development
Late fruiting	LG2 86 cM	meristem determinacy, flower development, flowering and embryo development
Speed of ripening (PCO1)	LG2 63 cM	vegetative to reproductive change, pollen and embryo development, post-embryonic development, anthocyanin and flavonoid biosynthesis
	LG3 17 cM	flowering, photoperiodism, flower and embryo development, response to light intensity
	LG3 56 cM	development of flower parts, photoperiodism, embryo development, anthocyanin accumulation
	LG5 40 cM	photoperiodism, embryo development
	LG3 94 cM	light signalling, response to light, flower and ovule (in June) development
Comparison between later and earlier scores (PCO2)	LG3 94 cM	flower development, light signalling, flowering, long day photoperiodism, embryo, flower, ovule development
	LG4 35 cM	flower development, embryo development, flowering, ovule development
	LG5 72 cM	floral meristem identity, flowering, flower development, pollen development, embryo development, post-embryo development
	LG6 3 cM	cell wall organisation
PCO4 Similar to PCO2	LG2 88 cM	meristem determinacy, flower development, flowering and embryo development
	LG3 76 cM	negatively regulating flowering and floral meristem cell accumulation, cell wall organisation and signalling processes, flowering, anther development embryo development
	LG5 17 cM	embryo development, negative regulation of flower development
	LG6 5 cM	embryo development

### Linking trait QTL to genome scaffolds and underlying genes

The GbS linkage map, in combination with the annotated draft raspberry reference genome, allowed the genomic regions that underlie the traits in Glen Moy to be identified and examined in terms of gene content, enabling the identification of genes with a potential role in the fruit developmental process (Table 5; Additional file 4: Table S1). QTL linked to the timing and rate of flower and fruit development identified 34 loci which contain several genes linked to flower, fruit, seed and embryo development, flower pollination and cell wall organisation. These loci also contained genes with roles in circadian regulation, signalling and timing of flowering to fruit and included transcription factors (Table 5; Additional file 4: Table S1). Map regions with no relation to ripening QTLs were explored for gene content (shown in Additional file 5: Table S2), confirming potentially ripening related genes identified were not random. One gene involved in multicellular organism development was identified in a region close to a QTL for root rot resistance and root diameter on LG6 however this is more likely to be involved in root lateral development rather than a having role in reproductive structure development thus is probably related to root growth during pathogen infection [23].

## Discussion

The Glen Moy x Latham segregating population has been used extensively to discover markers associated with many important raspberry traits [4, 7, 15, 22–30]. Prior to this study, Glen Moy x Latham linkage map comprised 439 markers, used to identify significant QTL associated with important raspberry agronomic characteristics (Table 2; [4]). GbS carried out here has enhanced the Glen Moy x Latham raspberry linkage map by adding 2348 high confidence SNP markers. The increase in marker coverage allowed us to define QTL more precisely and identify other significant loci not detected previously. To identify high confidence SNPs, we built the first draft genome assembly for red raspberry from the variety Glen Moy as a reference. This produced 360 Mbp of sequence ordered in 147,546 scaffolds that aligns with the recently published black raspberry genome, indicating that the red and black raspberry genomes are comparable ([21]; data not shown). We also identified SNPs through de novo assembly of the GBS tags from the parental and sibling lines, with 75% of the detected SNPs that had over 1000 sequence reads common to those identified from the reference genome assembly. A further 5823 de novo SNPs matched a reference *Pst*I site, but no SNP was called by the reference assembly method, indicating that there are other SNP markers that could potentially be added to this linkage map.

Examination of the SNP allele read counts showed that, in addition to the expected segregation patterns for biallelic SNPs, there were some configurations that could be more convincingly explained by the presence of a third, ‘null’, allele. This was also found by Russell et al. [35] in blackcurrant, who included the more informative ‘B’ allele from AO x AB and AB x AO SNPs in their map. For the revised raspberry map, both alleles were included for the loci with the ‘null’ configuration, and AO x BO SNPs were also mapped, in order to increase the number of mapped markers segregating in Glen Moy. These additional markers fitted on the linkage maps as well as markers without null alleles did.

The linkage map construction was challenging due to the high density of markers. Most methodological development for high-density mapping has been focused on crosses from homozygous parents, which are not suitable for crops such as raspberry. JoinMap’s regression mapping approach is reliable for such crosses, but it is extremely slow to run the usual three mapping rounds to place all markers. We used a rapid approach based on multi-dimensional scaling reported by Preedy and Hackett [44], which optimises a similar criterion to JoinMap’s regression mapping, and compared this to the sparser order obtained from two rounds of mapping with JoinMap. QTL mapping using the high-density map gave surprisingly irregular LOD profiles using the MapQTL software. A small simulation study indicated that the imbalance in information about the parental genotypes was most likely to be the cause of this, as much better results were obtained using a simulated blackcurrant map with equal numbers of markers from each parent. Genstat’s QTL mapping routine also gave poor results with the combination of imbalanced parental information and missing marker data. A hidden Markov model (HMM), which uses all the marker information on a chromosome to infer genotypic probabilities at each position, gave smoother profiles with more clearly defined peaks, and was used here.

We used a previously described dataset that measured the rate of fruit development from flower to over-soft fruit in the parental cultivars and 184 of their siblings grown in the field and under a protected polytunnel environment [7]. At that time the linkage map consisted of 243 markers, mostly AFLPs, with a total length of 843 cM. The QTL analysis performed here, with the addition of 2348 high confidence SNP markers, identified 34 significant loci for developing fruit from both field and sheltered grown raspberry. Many of these loci were found in the previous QTL analysis and confirmed the importance of these loci. The greatly increased number of markers allowed us to more clearly define the genetic position of the loci, as well as identify significant loci that were not described previously (Table 4). LG6 showed multiple QTLs between 3 and 9 cM for days to different stages of fruit development that were principally effects of the Latham allele and these were not all detected previously [7]. This region was close to a highly significant QTL for movement from a slow to rapid developmental transition at 3 cM found in the previous analysis [7] thus highlighting this region on LG6 as a key locus affecting the rate of fruit development. The ability to localise the segregating SNP markers to the newly created Glen Moy genome assembly identified underlying candidate genes associated with each locus. Arabidopsis gene orthologues underlying LG6 3–9 cM are annotated as having a role in cell wall organization, embryo development and cell division (Table 5). In this study the regions on LG3 and LG5 were also identified as important loci involved in the ripening process. A recent QTL analysis of fruit softening in the Glen Moy x Latham mapping population identified QTLs at the same positions on LG3 and LG5, with another significant loci at LG1 [30].

The range of QTL for different stages of fruit development has identified loci that are rich in genes relevant to flowering, fruit stages and the process of ripening (Table 5; Additional file 4: Table S1). Flowering time shifts in response to changes in climate and much is known about the genetic pathways regulating flowering and mechanisms underlying vegetative to flowering phase change [1, 2, 49]. However, the transition to flowering is regulated by a range of environmental and physiological cues that still need to be fully understood in perennial crops [50, 51]. In this study, a range of genes potentially involved, including a regulator of CO expression (FKF1, LG7 20 cM), FT, transport of FT (FTIP1), a gene regulating levels of FLC (EFL7) and COL9 regulating CO, FT and SOC1 were identified on LG3 at 8, 56 and 94 cM respectively (Additional file 4: Table S1). The QTL at LG2 at 57 cM found in days to red fruit mapped close to Gene H, a Myb transcription factor (Werewolf) which is a posttranscriptional regulator of FT [52, 53]. In other plant systems these are key components of the transition from vegetative to flowering state. RiMADS_01 was previously identified on LG5 as a potential candidate affecting vernalization [7] and is close to a QTL for green fruit and fruit set identified in this study. RiMADS_01 is similar to SVP modulating the timing of the developmental transition to flowering phase in response to temperature [54, 55] and in colder seasons RiMADS_01 was associated with earlier flowering. Close to RiMADS_01 is a region affecting developmental stages (PCO4), which included a range of genes regulating embryo development, flower development, meristem development, cell development and photoperiodism, including *Altered Meristem Program 1 (AMP1), Flowering Promoting Factor 1 (ATFPF1)* and *Reduced Vernalisation Response (VRN1)*.

The current model of fruit set implies that ovary growth is blocked before pollination and that auxin is a key regulator of ovary growth de-repression at fruit set [55, 56]. This study identified a range of auxin signalling and response gene homologs within the QTL including *ATCUL1, TOPLESS, NAC17, 12A, ARF6, ARF17, ATAVP1* (Additional file 4: Table S1). Other genes involved in ethylene synthesis, activation and signalling were identified within multiple QTL across LG3, LG5 and LG7. Raspberry is climacteric, but ethylene formation may have a minor role in raspberries that may be co-ordinated with auxin and ABA formation as part of the mechanism that regulates timing of ripening in different fruit species [57–59]. Finally, the self-incompatibility S locus lectin gene was found at a QTL on LG3. Gene *S* is common among the diploid *Rubus* spp., but domesticated forms are self-compatible due to a mutation.

Recognising the processes controlling fruit development and ripeness in raspberry is essential to understand important agronomic and fruit quality characteristics. Time to flowering, time to fruit set and ripening, fruit flavour, colour, shape and softening are all important characteristics that affect the timing and quality of the produce and are under selection by fruit breeders. Environmental differences and changes in agronomical practices are affecting how these characteristics develop and are leading to difficulties in farming practice. Saturated linkage maps and QTL analysis of segregating populations are important methods for dissecting some of these more complex traits and identifying markers used in marker assisted breeding programs. The establishment of this new, high-density, raspberry GbS linkage map, linked to genome scaffolds underpins the study of a large number of fruit development traits by accessing genes linked to genetic markers which can be validated in other populations or through gene expression studies. This GbS map enables re-analysis of multiple phenotypic data sets of key raspberry traits and relates to genome scaffolds and therefore candidate genes for analysis.

## Conclusion

We have established a high-density GbS map in red raspberry that allows QTL to be identified more precisely. We also established a draft genome sequence for Glen Moy that aided the development of the GbS map and also allows to directly assign SNP marker information to the Glen Moy genome scaffolds. We used these resources to perform a more precise QTL analysis of developing fruit to understand the genetic control of fruit ripening traits and identify candidate genes. Genes associated with QTL will be examined along with gene expression changes across fruit development to identify the key regulatory control across the process of development.

## Additional files

Additional file 1: Appendix A. Workflow for the linkage mapping procedure. (DOCX 15 kb)

Additional file 2: Appendix B. Fitting a hidden Markov model (HMM) to obtain QTL genotype probabilities from marker data. (DOCX 24 kb)

Additional file 3: **Figure S1.** QTL profiles from simulated data. S1(a)-(c) map a QTL simulated on 67 cM on the map of raspberry LG2 using MapQTL, Genstat and a HMM respectively. S1(d)-(f) map a QTL of the same size, simulated at 29 cM, on the blackcurrant linkage map (where there is a similar amount of marker information for each parent). (DOCX 39 kb)

Additional file 4: **Table S1.** Genome scaffolds within 2 cM of most significant marker containing genes with a potential role in ripening related developmental processes. This table examines genes within the QTL regions which may have a role in developmental processes leading to fruit ripening. (DOCX 30 kb)

Additional file 5: **Table S2.** Two non-related regions with associated scaffolds within 2 cM and any potential developmentally/ripening related genes. This table examines regions not identified in ripening to ensure the ripening related genes are not chance associations. (DOCX 17 kb)

## Abbreviations

BAM: Binary Alignment/Map
cv: Cultivar
FLG: *Fragaria* linkage groups
GbS: Genotyping by sequencing
HMM: Hidden Markov model
LOD: Logarithm of the odds ratio
MDS: Multi-dimensional scaling
PCOP: rincipal co-ordinate analysis
PCRP: olymerase chain reaction
QTL: Quantitative trait loci
SNPs: Single nucleotide polymorphisms
