# Supplementary material for: Enhancement of Glen Moy x Latham raspberry linkage map using GbS to further understand control of developmental processes leading to fruit ripening
Source: BMC Genet. 2018 Aug 15;19:59. doi: 10.1186/s12863-018-0666-z (PMC6094467; doi:10.1186/s12863-018-0666-z)
Supplement: Supplementary file 1 — Appendix A. Workflow for the linkage mapping procedure. (DOCX 15 kb) [file 12863_2018_666_MOESM1_ESM.docx]

**Appendix A Workflow for linkage mapping**

1. Filter SNPs from freeBayes using quality score and mean read counts
2. Marker regression mapping of allele proportions onto raspberry linkage map [4] to allocate SNPs to chromosomes
3. Classify SNP allele read counts to genotypes using functional regression approach
4. Filter to exclude SNPs with unexpected ratios, given the parents. SNPs showing a 2:1:1 ratio compatible with an AO x AB or AB x AO genotype were retained as separate A and B alleles.
5. For each linkage group
   1. Combine SNP markers with markers from previous map
   2. Within JoinMap, eliminate markers with severe distortion, many missing values or duplicate markers
   3. Calculate recombination fraction and LOD score for all pairs
   4. Run MDS analysis, using LOD weighting and a two-dimensional fit, to identify and omit outliers, and so to obtain initial GbS map
6. Add any extra SNPs that appear to segregate as AOxBO markers (keeping A and B alleles together) to each linkage group
7. Repeat linkage mapping step 5, comparing different MDS stress measures
8. Analyse each map using HMM to find regions with many double recombinants. Rerun MDS if necessary
