# Supplementary material for: Enhancement of Glen Moy x Latham raspberry linkage map using GbS to further understand control of developmental processes leading to fruit ripening
Source: BMC Genet. 2018 Aug 15;19:59. doi: 10.1186/s12863-018-0666-z (PMC6094467; doi:10.1186/s12863-018-0666-z)
Supplement: Supplementary file 2 — Appendix B. Fitting a hidden Markov model (HMM) to obtain QTL genotype probabilities from marker data. (DOCX 24 kb) [file 12863_2018_666_MOESM2_ESM.docx]

**Appendix B: Fitting a hidden Markov model (HMM)**

A hidden Markov model (HMM) has a series of states, forming a path. Transition probabilities model the transitions from one state to another. From each state, symbols are emitted, modelled by emission probabilities. Only the symbols are observable, and the aim is to infer the path of states from the observed symbols.

Denote the path of states as {π_i_, *i*=1…*K*} and the sequence of observed symbols as {*x_i_*, *i*=1…*K*}. The transition probability between states is given by

$$a_{kl}=p(\pi_{i}=l|\pi_{i-1}=k)$$

and is independent of the states at earlier times. The emission probability is given by

$$e_{k}(b)=p(x_{i}=b|\pi_{i}=k)$$

and depends on the state of the hidden variable at time *i*.

To apply this to an offspring from a cross between parents with genotypes AB x CD, consider the four possible offspring genotypes AC, AD, BC, BD to be the unobservable states at SNP positions 1 to *K* along the chromosome, where positions here replaced the usual HMM ‘times’. Associated with each genotype is an indicator *s_ik_*, equal to 1 if the offspring marker phenotype at position *i* can be obtained from genotype *k*, and 0 otherwise. The observed symbols are a sequence of *K* symbols ‘y’, indicating correct matches between the genotypes and the phenotypes. The set of emitted symbols from each state is {y,n}. If the marker data is regarded as error-free, the emission probabilities are

$$\begin{matrix} p(x_{i}='y'|\pi_{i}=k)=s_{ik} \\ p(x_{i}='n'|\pi_{i}=k)=1-s_{ik} \end{matrix}$$

However the marker data may have been scored incorrectly, with probability λ. The emission probabilities therefore become

$$\begin{matrix} p(x_{i}='y'|\pi_{i}=k)=s_{ik}(1-\lambda)+(1-s_{ik})\lambda\\ p(x_{i}='n'|\pi_{i}=k)=s_{ik}\lambda+(1-s_{ik})(1-\lambda) \end{matrix}$$

The transition probabilities depend on the distance between markers, and the number of recombinations (0, 1 or 2) to move between two states. The transition probabilities can be written as

$$a_{kl}=p(\pi_{i}=l|\pi_{i-1}=k)=r_{i-1,i}^{d_{kl}}(1-r_{i-1,i})^{2-d_{kl}},d_{kl}=0...2$$

where *r* is the recombination fraction between positions *i* -1 and *i*, and *d_kl_* is the number of recombinations between those states. From the HMM, we need to estimate the probabilities of each of the different genotype states along the path,

$$\{p(\pi_{i}=k{}_{i}|x),i=1...K\}$$

These are known as the posterior state probabilities and can be solved by use of recursive algorithms, the forward algorithm and the backward algorithm [60]. The scaling method for these algorithms, as described there, was used to avoid computing underflow errors. These give the posterior state probabilities of each genotype at each position, along with the overall probabilities of the sequence *p(x)*. This gives genotype probabilities at the SNP positions along the chromosome.

Hidden Markov models were used in two contexts in this paper. For checking the reconstruction of the state to see if there were high levels of recombinations at any positions, the error probability λ was set at zero. For QTL mapping, the error probability was set at 0.01. To map QTLs along the entire chromosome also requires QTL genotypes to be estimated between the SNP positions. Here linear interpolation was used to interpolate the probabilities with a 1 cM spacing.
