# Supplementary figures and images for: Enhancement of Glen Moy x Latham raspberry linkage map using GbS to further understand control of developmental processes leading to fruit ripening
Source: BMC Genet. 2018 Aug 15;19:59. doi: 10.1186/s12863-018-0666-z (PMC6094467; doi:10.1186/s12863-018-0666-z)

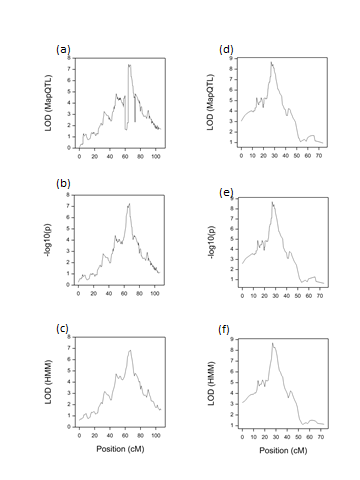

Supplement: Supplementary file 3 — Figure S1. QTL profiles from simulated data. S1(a)-(c) map a QTL simulated on 67 cM on the map of raspberry LG2 using MapQTL, Genstat and a HMM respectively. S1(d)-(f) map a QTL of the same size, simulated at 29 cM, on the blackcurrant linkage map (where there is a similar amount of marker information for each parent). (DOCX 39 kb) [file 12863_2018_666_MOESM3_ESM.docx]
