# Supplementary material for: Enhancement of Glen Moy x Latham raspberry linkage map using GbS to further understand control of developmental processes leading to fruit ripening
Source: BMC Genet. 2018 Aug 15;19:59. doi: 10.1186/s12863-018-0666-z (PMC6094467; doi:10.1186/s12863-018-0666-z)
Supplement: Supplementary file 4 — Table S1. Genome scaffolds within 2 cM of most significant marker containing genes with a potential role in ripening related developmental processes. This table examines genes within the QTL regions which may have a role in developmental processes leading to fruit ripening. (DOCX 30 kb) [file 12863_2018_666_MOESM4_ESM.docx]

**Additional file 4.**

Table S1. Genome scaffolds within 2 cM of most significant marker containing genes with a potential role in ripening related developmental processes.

| Trait QTL and any other trait QTL overlap | Linkage group | Scaffolds in region (size bp) | TAIR | Name and potential role in development |
| --- | --- | --- | --- | --- |
| **open flowers**  **(field)**  Overlaps QTL for PCO4 (field) | LG2 85cM | 976 (62739)  541 (56716)  541  7893:454C89709_AGL* (7420)  3294 (26461)  5782 (18051)  461 (61634)  3525 (17915)  22199 (1864) | AT5G45840  AT5G45800  AT4G18650  AT4G18960  AT5G45750  AT1G78580  AT1G29950  AT4G18830  AT4G35540 | LLR-RLK pollen tube guidance  MEE62, embryo development  Maternally expressed imprinting gene  AG, carpel, flower& stamen development, floral organ identity  RAB GTPase, cell wall biogenesis  TPS1, cell division, embryo development  bHLH, transcription factor  OFP5, embryo sac development  PTF2, embryo development, pollen germination |
| **open flowers**  **(field)**  Overlaps QTL PCO1 (field)  Close to QTL for Red fruit trait (field & polytunnel) | LG3 17cM | 88 (169528)  88  88  5426 (11913)  408:ERubLRSQ13* (65601)  1896 (38052)  1349 (34099)  392 (135395)  2144 (25806)  95 (160529)  201 (99814)  5379 (24932)  863 (52898)  581 (54524)  341 (71475)  408 (65601)  408 | AT3G51630  AT2G40610  AT4G32010  AT3G05120  AT3G02780  AT4G33990  AT3G21510  AT3G51550  AT1G08970  AT5G01600  AT3G08850  AT4G02570  AT3G04580  AT3G04340  AT3G51770  AT5G56270  AT3G02730 | WNK family, flowering, photoperiodism  EXPA8, cell wall organisation, cell growth  HS12-like, positive regulation seed development  GA insensitive, floral organ development, signalling  IPP2, flower development  EMB2758, embryo development  AHP1, embryo sac development, signalling  FER, post-embryonic development, pollen tube reception  NF-YC, photomorphogenesis, signalling  FER1, flower development  RAPTOR1, cell growth, embryo development  ATCUL1, auxin activated signalling, embryo development  EIN4, ethylene signalling  EMB2458, embryo development  ETO1, signalling, post-embryonic development  WRKY, cell polarity, pollen development  ATF1, response to light intensity |
| **open flowers**  **(polytunnel)**  Overlaps QTL % open flowers (Polytunnel)  Close to QTL for PCO4 (field) and set (polytunnel) | LG3 62cM | 3246 (19060)  2268 (24824)  418 (84987)  499 (89619)  4425 (14711)  352 (70625)  3246 (19060) | AT1G15750  AT2G42560  AT5G25190  AT2G21540  AT2G30900  AT3G42170  AT1G15750 | TOPLESS, embryogenesis, response to auxin  LEA25, embryo development  ERF, ethylene activated signalling  SEC14-LIKE, flower development  TBL43, cell wall organisation  DAYSLEEPER, post-embryonic development TOPLESS, embryo development, response to auxin, signalling |
| **open flowers**  **(field)**  overlaps QTL for PCO1 (field) | LG5  40cM | 1011 (39457)  3358 (72592)  3358 (22715)  571 (55260)  1483 (44388)  355 (70324)  4745 (13740) | AT4G16770  AT5G46940  AT4G17615  AT1G04635  AT4902570  AT5G47010  AT4G17500 | 2OG, flanonoid biosynthetic process  PME-I, catalytic activity  ATCBL1, signalling, pollen tube growth  EMB1687, embryo development  Embryo development, signalling  ATUPF1, Long day photoperiodism, signalling  ERF, ethylene activated signalling, cell division |
| **% open flowers (polytunnel)**  Overlaps QTL for bud break/open (polytunnel)  Close to QTL set (polytunnel) and PCO4 (field) | LG 3 66cM | 149 (148147)  4316 (15056)  784 (53872)  352 (70625)  1205 (46721)  367 (68950)  367  693 (49122)  693 | AT1G80070  AT5G06950  AT1G30950  AT3G42170  AT1G79860  AT5G11590  AT3G16240  AT2G41945  AT1G11330 | EMB, embryo development  AHBP-1B, bZIP, transcriptional control, signalling  UFO, flower development  DAYSLEEPER, post-embryonic development  MEE64, embryo development, pollen tube growth, regulation of pollen tube growth  ERF/AP2, ethylene activated signalling, transcription  AQP1, water channel  RED1, embryo development  S-locus lectin, pollen recognition |
| **Fruit set**  (polytunnel)  Overlaps QTL PCO4 (field) and Close to fruit set (field) and % open flowers (polytunnel) | LG 3 76cM | 4615 (27543)  1320 (38675)  922 (83608)  922  28: Ri9022* (392062)  30: Ri 9022* (383020)  33: Ri9022* (366235)  41: Ri9022* (313000)  42: Ri9022 (313098)  43: Ri9022 (302968) | AT1G79730  AT1G71930  AT1G79750  AT3G16350  AT2G06255  AT1G17440  AT5G35550  AT4G28190  AT3G01040  AT1G72570 | EFL7, negative regulation of flowering by elevating FLC  NAC-17, cell wall organisation, xylem formation, response to auxin & brassinosteroid  NADP-Malic enzyme, embryo development ending in seed dormancy  MyB-like, auxin response  ELF4, circadian rhythm  CKH1, ethylene activated signalling, jasmonic acid signalling  ATMYB123, proanthocyanin accumulation in seed  ULT, negative regulator of floral meristem cell accumulation  GAUT13, pollen tube growth  Integrase, ethylene activated signalling, multicellular organism development |
| **Fruit set**  **(field)**  Overlaps QTL green fruit (field)  Adjacent to QTL PCO2 (field) and close to fruit set (polytunnel) | LG 3 102cM | 4 (654795)  4  4  4  294 (78274)  52 (255735)  52  52  162 (111302)  162  734 (47642)  734  734  65 (210475)  65  65  858 (43514)  858 | AT1G05230  AT1G62360  AT5G64610  AT2G05710  AT5G35700  AT2G31340  AT1G05010  AT1G50640  AT2G04842  AT5G35750  AT1G80070  AT4G03210  AT5G12210  AT4G28210  AT4G28190  AT2G31180  AT4G03240  AT2G19130 | HDG2, maintenance of floral organ identity  BUM, carpel development, SAM formation during embryogenesis  Aconitase  HAM1, photoperiodism, flowering  FIM2, pollen germination, pollen tube growth  EMB1381, embryo development ending in seed dormancy  ACO4, ethylene biosynthetic process, fruit ripening  ATERF3, ethylene signalling  EMB2761, embryo development ending in seed dormancy  AHK2, regulation of flower development  EMB14, embryo development  XTH9, cell wall biogenesis  ATRGTB1, pollen development  EMB1923, embryo development ending in seed dormancy  ULT, regulator of floral meristem cell accumulation, flower number  ATMYB14, cell differentiation, response to ethylene, jasmonic acid  ATFH, embryo development  S-locus lectin, pollen recognition |
| **Fruit set**  **(field)**  Overlaps QTL green fruit (field) | LG 5 1cM | 376 (68183)  1741 (41801)  1741  2571 (28920)  1110 (43353)  3614 (28001) | AT5G64610  AT1G02680  AT2G18390  AT1G68090  AT1G77850  AT1G65690 | HAM1, photoperiodism, flowering  TAF13, embryo, endosperm & seed development  ARF like, embryo and endosperm development  ANN5, pollen development & germination  ARF17, signalling, cell wall development pollen development, regulates auxin response genes  NDR1/HIN1-Like, seed development, ABA signalling |
| **Fruit set** (field)  Overlaps QTL green fruit (field)  Adjacent to QTLs for PCO4 (field), PCO2 (field), Green (field) | LG 6  9cM | 122 (152105)  122 | AT5G37680  AT5G42390 | ARF-like, cell division  SPP, embryo development ending in seed dormancy |
| **Fruit set**  (field) | LG 7 20cM | 21 (424263)  21  21  21  21  3 (665954)  3  3  3  3  3  3  3  3  3  3  3  3  20 (428130)  104 (152507)  104  104  16 (454659)  16  16  16  38 (336821)  38  38  38  19 (437302)  19  19  19  19  19  19  19  19  19  19  19  19  19  19  19 | AT1G48920  AT5G23320  AT3G20800  AT5G23130  AT5G23150  AT1G59940  AT2G38110  AT1G10430  AT1G60420  AT1G60220  AT1G23540  AT1G24170  AT1G70000  AT5G62620  AT2G38280  AT2G16910  AT1G70170  AT1G10510  AT3G42960  AT1G68050  AT1G67960  AT4G08850  AT1G23420  AT4G38620  AT4G02570  AT2G01290  AT2G01620  AT2G01570  AT2G01730  AT1G60460  AT2G02820  AT1G69500  AT2G02970  AT1G26830  AT2G03050  AT2G03150  AT1G26945  AT5G39660  AT2G03060  AT1G26770  AT5G15310  AT3G47620  AT1G26840  AT1G14360  AT1G69490  AT2G02990 | ATNUC-L1, development  ATICMTA, development, flower development  Rcd1-like, development  LysM domain, cell wall  Enhancer of AG-4, anthocyanin accumulation, cell differentiation, regulation of flowering, transition to flowering, activator of FLC expression, enhances AG function  ARR3, Circadian rhythm  ATGPAT6, cutin biosynthetic process, flower development  PP2A-2, signalling  ATNRX1, pollen tube growth, guidance, regulation of growth  OTS1, flowering time regulation  ATPERK12, fertility, development  GATL8, cell wall organisation, pectin biosynthetic process  MYBD, anthocyanin biosynthesis  GALT6, Seed coat development, negative regulation of leaf senescence  ATAMPD, embryo development ending in seed dormancy  AMS, bHLH, anther wall development, pollen development  MMP, development, regulation of photoperiodism  EMB2004, embryo development ending in seed dormancy  ASD, flower development  FKF1, regulates CO expression, flowering  POD1, pollen tube guidance, embryo development  MDIS-1IRLK2, pollen tube guidance  INO, YABBY, ovule development  ATMYB4, cell differentiation  ATCUL1, embryo development ending in seed dormancy, ethylene activated signalling, auxin activated signalling, jasmonic acid acid activated signalling  RPI2, Vegetative to reproductive phase transition of meristem, flowering time control  MEE11, embryo development ending in seed dormancy  RGA, fruit and flower development, signalling  Embryo Sac Developmental Arrest 26, development of embryo  MTOPVIB, embryo sac morphogenesis  ATMYB88, embryo sac development  Cytochrome P450, pollen exine formation  APY6, anther dehiscence, pollen exine formation  ATCUL3, embryo development ending in seed dormancy, endosperm development, flowering  EMB93, embryo development ending in seed dormancy  EMB1579, embryo development ending in seed dormancy  KDR, bHLH involved in light signalling  CDF2, flower development  AGL30, pollen maturation, pollen tube growth  ATEXPA10, cell wall loosening and modification  MYB16, cell morphogenesis  ATTCP14, cell proliferation, inflorescence development  ATORC6, DNA replication, pollen development  ATUTR3, embryo sac development  ANAC029, embryo development ending in seed dormancy  ATRNS1, anthocyanin biosynthesis, aging |
| **Green**  (field) | LG 3  49cM | 1998 (47377)  2945 (20523) | AT5G02800  AT2G37260 | CDG1-like, brassinosteroid signalling, plant growth  ATWRKY44, seed coat development |
| **Green** (field)  Overlaps QTL for fruit set (field) | LG 3  105cM | 65 (210475)  65  65  858 (43514)  858  734 (47642)  734  734  4 (654795)  4  4  4  4  4  4  182 (104634)  509 (59042)  43 (302968)  43  43 | AT4G28210  AT4G28190  AT2G31180  AT4G03240  AT2G19130  AT1G80070  AT4G03210  AT5G12210  AT3G04090  AT5G54650  AT2G32280  AT4G15180  AT1G62360  AT2G46410  AT1G05190  AT3G02280  AT3G02150  AT2G37630  AT1G13280  AT3G02380 | EMB1923, embryo development ending in seed dormancy  ULT, multicellular organism development, floral meristem determinacy, regulation of inflorescence meristem growth  ATMYB14, cell differentiation, response to ethylene, response to auxin  ATFH, embryo development  S-locus lectin, recognition of pollen  EMB14, embryonic development  XTH9, cell wall biogenesis, cell wall organisation  ATRRGTB1, pollen development  SIP1, aquaporin, transport  FH5, endosperm development, seed morphogenesis  VCC, embryo provasculature development  ATXR3, carpel development, post-embryonic development  BUM, carpel development, SAM formation during embryogenesis  CPC, cell differentiation, epidermal cell differentiation  EMB2394 embryo development ending in seed dormancy  ATTAH18, embryo development ending in seed dormancy  A-PTF1, cell differentiation  ATMYB91, cell division, cell differentiation  AOC4, jasmonic acid biosynthesis  ATCOL2, flowering time |
| **Green** (field)  Overlaps QTL for fruit set (field) | LG 5 1cM | 376 (68183)  1741 (41801)  1741  1741  2571 (28920)  1110 (43353)  3614 (28001)  10160:ErubendoSQ07_P15XL* (11060) | AT5G64610  AT3G62580  AT1G02680  AT2G18390  AT1G68090  AT1G77850  AT1G65690  AT1G78060 | HAM1, regulation of flowering period  LEA protein, biological process  TAF13, embryo development ending in seed dormancy, endosperm development  ARF-Like 2, embryo development ending in seed dormancy, embryo sac cellularisation, endosperm development  ANN5, pollen development, pollen germination, pollen tube growth  ARF-17, development, cell wall, pollen wall assembly  NDR1/HIN1-Like, seed development  BXL7, cell wall, carbohydrate metabolism |
| **Green** (field)  Overlaps QTL fruit set (field)  Adjacent to QTLs for PCO4 (field), PCO2 (field), Green (field) | LG 6 9cM | 122 (152105)  122 | AT5G37680  AT5G42390 | ARLA1A, cell cycle, cell division  SPP, embryo development ending in seed dormancy |
| **Green/red**  (field)  Overlaps QTL red fruit (field)and overlaps QTL for PCO2 (field) | LG 4 39cM | 702 (80412)  702  452 (95458)  39968:454C207_PG* (963) | AT5G44120  AT4G28520  AT3G04400  AT2G41850 | ATCRA1, seed maturation  CRC, embryo development ending in seed dormancy, seed maturation  EMB2171, embryo development ending in seed dormancy  ADPG2, cell wall modification, floral organ abscission, fruit dehiscence |
| **Green/red** (field)  Overlaps QTL PCO2 (field), and QTL PCO4 (field) Adjacent to QTL fruit set (field) and green fruit (field) | LG 6 3cM | 2652:454Cl6475_Arabino* (38682) | AT4G19410 | PAE7, cell wall organisation |
| **Green/red** (polytunnel) | LG6 46cM | 4722 (13801) | AT3G06350 | EMB3004, embryo development ending in seed dormancy |
| **Red** (field) adjacent to QTL bud break/open flowers (field) and QTL PCO1 (field) | LG 2 57 | 1694 (29803)  789 (45768)  2826 (21273)  299 (150130)  299  299 | AT3G20440  AT4G02570  AT3G42170  AT5G23960  AT4G27060  AT5G17410 | BE1, embryo development ending in seed dormancy, post-embryonic development  ATCUL1, embryo development ending in seed dormancy, auxin signalling & response  DAYSLEEPER, post-embryonic development  ATTPS21, floral volatile generation  CN, unidimensional cell growth  Spc97, gametophyte development |
| **Red** (field & polytunnel)  Close to QTL for bud break/open flowers (field) and PCO1 trait (field) | LG 3 8-9cM | 408 (65601)  408  5341 (15959)  502 (64533)  502  301 (81553)  11 (500530)  11  11  11  11  11  11  11  11  5426 (11913)  1134 (108488)  1134  1636 (51204)  39 (322430)  2 (708840)  2  2  2  1896 (38052) | AT5G16460  AT5G56270  AT3G04690  AT2G02240  AT3G07020  AT3G22780  AT3G62290  AT4G37750  AT1G56710  AT4G10710  AT1G09530  AT5G48670  AT3G05910  AT1G09540  AT4G37750  AT3G05120  AT1G65480  AT5G16260  AT3G06350  AT5G26742  AT1G15690  AT3G05620  AT3G05530  AT2G47470  AT4G33990 | SEIPIN1, seed development  WRKY, cell polarity, embryo development  ANX1, pollen tube growth  MEE66, embryo development ending in seed dormancy  SGT, seed development  ATTSO1, floral organ morphogenesis, regulation cell division, regulation of meristem structural organisation  A1E, Cell expansion, cellulose production  ANT, cell proliferation, flower development, gamete generation, apical meristem identity  Pectin lyase, cell wall organisation  SPT16, binds to FLC promoter  PAP3, signalling, anthocyanin metabolism  AGL80, cell development  PE, cell wall organisation  MYB 61, seed coat development, stomatal movement  ANT, control of cell proliferation, organ morphogenesis  GID1A, floral organ morphogenesis, GA signalling, ovule develpment  FT, cell differentiation, flower development, regulation of flower development, stomatal movement  ELF9, negative regulation of flower development  EMB3004, embryo development ending in seed dormancy  EMB1138, embryo development ending in seed dormancy  ATAVP1, auxin transport, transmembrane electrochemical gradient, water deprivation  Plant invertase, cell wall modification  ATS6A2, embryo sac development, pollen development  ATPDI11, embryo sac development, pollen development, endosperm development, embryo development ending in seed dormancy  EMB2758, embryo development ending in seed dormancy |
| **Red** (field)  Overlaps QTL Green/Red (field) and close to PCO2 (field) | LG 4 42cM | 1289 (34931)  700 (48833)  452 (95458)  66107:454207_PG* (592) | AT5G43940  AT3G07020  AT3G04400  AT1G56710 | AHD2, seed development  SGT, seed development  EMB2171, embryo development ending in seed dormancy  Pectin lyase, cell wall organisation |
| **Late ripening (field)**  Overlaps PCO4 (field) | LG 2 88cM | 22199 (1864)  3294 (26461)  3525 (17915)  541 (56716)  541  579 (60305)  163 (117274)  163  163  163 | AT4G35540  AT5G45750  AT4G18830  AT5G45840  AT5G45800  AT1G28520  AT4G28500  AT2G33860  AT5G60910  AT1G28420 | PTF2, pollen germination, embryo development ending in seed dormancy  RAB GTPase, cell wall biogenesis  OFP5, embryo sac development  MDIS1, pollen tube guidance  MEE62, embryo development ending in seed dormancy  ATVOZ1, flowering, long-day photoperiodism  NAC, multicellular organism development, cell wall biogenesis  ARF3, floral meristem determinacy, flower development, auxin signalling  AGL8, flower development, fruit development, meristem maintenance  RINGLET 2, vegetative to reproductive phase transition |
| **PCO1**  **(field)** | LG 2 63cM | 105 (152123)  105  2568 (22781)  240 (198871)  240  240  440  60 (233038)  60  264 (83572) | AT3G42170  AT3G15370  AT1G53329  AT1G78579  AT1G17020  AT3G14750  AT5G46760  AT1G53160  AT5G53860  AT3G15150 | DAYSLEEPER, post-embryonic development  ATEXP12, cell wall loosening, unidimensional growth  ATTLP7, pollen development, embryo development  ATRHM1, flavonoid biosynthesis  Flower development, cell differentiation  MYC3, anthocyanin biosynthesis  SPBL, vegetative change, flower development  EMB64, embryo development ending in seed dormancy  ATMMS21, meristem maintenance |
| **PCO1** (field)  Co-locates with open (field) | LG 3 16cM | 88 (169528)  88  88  5426 (11913)  408:ERubLRSQ12.4A04 DMQ& ERubLRSQ13.2C12 IPPI * (65601)  1896 (38052)  1349 (34099)  392 (135395)  2144 (19641)  95 (160529)  201 (99814)  5379 (24932)  863 (52898)  581 (54524)  341 (71475)  408 (65601)  408 | AT3G51630  AT2G40610  AT4G32010  AT3G05120  AT3G02780  AT4G33990  AT3G21510  AT3G51550  AT1G08970  AT5G01600  AT3G08850  AT4G02570  AT3G04580  AT3G04340  AT3G51770  AT5G56270  AT3G02730 | WNK family, flowering, photoperiodism  EXPA8, cell wall organisation, cell growth  HS12-like, positive regulation seed development  GA insensitive, floral organ development, signalling  IPP2, flower development  EMB2758, embryo development  AHP1, embryo sac development, signalling  FER, post-embryonic development, pollen tube reception  NF-YC, photomorphogenesis, signalling  FER1, flower development  RAPTOR1, cell growth, embryo development  ATCUL1, auxin activated signalling, embryo development  EIN4, ethylene signalling  EMB2458, embryo development  ETO1, signalling, post-embryonic development  WRKY, cell polarity, pollen development  ATF1, response to light intensity |
| PCO1 (polytunnel) | LG 3 56cM | 4803 (13567)  5888 (17132)  73 (197693)  3264 (18968)  9301 (11756)  1901:454Cl8848Cl_ZFP8* (27653)  674 (49896)  1340 (43120)  4414 (26528)  418 (84987)  564 (59473)  564  564  879 (42803)  564 (59473)  1315 (34611)  5935 (10756)  235 (99790)  1407 (33361)  8535 (14749)  4515 (18463)  176 (106226)  2070 (31735)  879 (42803)  16234 (2689) | AT2G36910  AT5G06850  AT1G10180  AT3G57800  AT4G20970  AT2G41940  AT2G41970  AT3G57670  AT1G23290  AT5G25190  AT3G44735  AT5G04560  AT5G03730  AT3G08590  AT3G44735  AT3G04100  AT5G65790  AT2G36880  AT2G36830  AT2G36190  AT5G03700  AT4G31400  AT5G03790  AT3G08590  AT3G22880 | ABCB1, Stamen development, anthocyanin accumulation, auxin signalling  FTIP1, flower development, long day photoperiodism, vegetative to reproductive phase, transport of FT  EXO84C, acceptance of pollen  BHLH  BHLH  ZFP8, multicellular organism development, GA signalling  MARIS, pollen tube growth  NTT, pollen tube growth, cell fate  RPL27A, flower development, post-embryonic development  ESE3, ethylene signalling  ATPSK3, cell differentiation, multicellular organism development  DEMETER, embryo development ending in seed dormancy, gene imprinting  ATCTR1, ethylene signalling, vegetative to reproductive transition  IPGAM2, pollen development, stomatal movement  ATPSK3, cell differentiation, multicellular organism development  AGL57, embryo development  ATMYB68, petal differentiation  MAT3, pollen tube growth  TIP1, water channel  ATCWI4, nectar secretion  D-mannose binding lectin, pollen recognition  ATCIF7, anther development, embryo development, embryo sac development  ATHB51, floral meristem determinacy, transition vegetative to reproductive phase  IPGAM2, pollen development  ARLM15, meiotic recombination |
| **PCO1** (field)  Overlaps QTL bud break/open flowers (field) | LG 5 40cM | 1011 (39457)  335 (72592)  3358 (22715)  571 (55260)  1483 (44388)  5041 (28006)  4745 (13740) | AT4G16770  AT5G46940  AT4G17615  AT1G04635  AT4G02570  AT4G37680  AT4G17500 | 2OG, flanonoid biosynthetic process  PME-I, catalytic activity  ATCBL1, signalling, pollen tube growth  EMB1687, embryo development  CUL1, auxin activated signalling, embryo development  HHP4, hormone response  ERF, ethylene activated signalling, cell division |
| **PCO2** (field)  Adjacent to QTL set (field & tunnel) | LG 3 94cM | 664 (50302)  936 (41068)  4025:454C2985_PSY* (15938)  676 (62949)  835 (59476)  3183 (36453)  76 (211143)  76 | AT1G30330  AT5G08560  AT5G17230  AT1G53580  AT3G22170  AT3G07650  AT5G35770AT5G48160 | ARF6, flower development, auxin signalling  ATWDR26, response to ethylene, auxin, light  PSY, carotenoid biosynthesis  ETHE1, embryo development ending in seed dormancy  CPD45, light signalling, circadian rhythm  COL9, flowering, long-day photoperiodism, downregulated CO, FT and SOC1  STERILE APETALA, flower and ovule development  OBE2, embryo development ending in seed dormancy |
| PCO2 (field)  Overlaps QTL green/red (field) adjacent to red (field) | LG 4 35cm | 537 (57009)  4014 (24985)  4014  2481 (23249)  318 (85856) | AT3G22490  AT3G54650  AT5G55300  AT2G47990  AT3G63080 | ATRAB28, embryo development ending in seed dormancy  FBL17, embryo development  TOP1, embryo development, flower morphogenesis  EDA13, embryo sac development  ATGPX5, embryo development ending in seed dormancy |
| **PCO2 (field)** | LG 5 72cM | 374 (68504)  1057:454c5803* (74573)  1057  10 (544111)  10  10  10  10  236 (89031)  236  236  506 (59317)  506  12 (497234)  12  12  12  12  12  12  12  638 (51685)  647 (51140) | AT2G46770  AT5G07990  AT1G18640  AT1G49040  AT5G23040  AT5G08170  AT3G18550  AT1G53330  AT1G32640  AT3G50790  AT3G61880  AT1G69120  AT5G17400  AT1G18370  AT5G41990  AT3G13540  AT1G64520  AT4G10760  AT1G68320  AT1G63910  AT5G46290  AT5G07290  AT1G32930 | ANAC043 embryo development ending in seed dormancy, anther dehiscence, fruit dehiscence, cell wall development  CYP75B1 flavonoid biosynthesis, auxin response  PSP, embryo development, pollen development  SCD1, flower morphology  CDF1, embryo development ending in seed dormancy  EMB1873, embryo development ending in seed dormancy  ATBRC1, arrests axillary bud growth  PPR, embryo development, fruit development  ATMYC2, signalling, flavonoid biosynthesis, JA signalling  Esterase, embryo development ending in seed dormancy  CYP78A9, fruit development  AGL7, floral meristem determinacy, flower development, floral meristem identity, flowering time, regulates SVP, SOC1 and AGL24  ER-ANT1, seed development  HIK, pollen development, embryo sac cellularisation  ATWNK8, photoperiodism, flowering, flower development  ATMYB5, seed coat development, trichome differentiation  12A, flower development, post-embryonic development, response to auxin  EMB1706, embryo development ending in seed dormancy, mRNA methylation  ATMYB62, cell differentiation, GA response  ATMYB103, cell differentiation, cell wall biogenesis  KAS1, embryo development  AML4, meristem development, regulation of growth  ATGALT31A, embryo development ending in seed dormancy |
| **PCO2 (field)**  Overlaps QTL Green/Red (field), adjacent to QTL PCO4 (field) | LG 6 3cM | 2652:454Cl6475* (38682) | AT4G19410 | PAE7, cell wall organisation |
| PCO4 (field)  Adjacent to QTL open (field) | LG 2 88cM | 22199 (1864)  3294 (26461)  3525 (17915)  541 (56716)  541  579 (60305)  163 (117274)  163  163  163 | AT4G35540  AT5G45750  AT4G18830  AT5G45840  AT5G45800  AT1G28520  AT4G28500  AT2G33860  AT5G60910  AT1G28420 | PTF2, pollen germination, embryo development ending in seed dormancy  RAB GTPase, cell wall biogenesis  OFP5, embryo sac development  MDIS1, pollen tube guidance  MEE62, embryo development ending in seed dormancy  ATVOZ1, flowering, long day photoperiodism  NAC, multicellular organism development, cell wall biogenesis  ARF3, floral meristem determinacy, flower development, auxin metabolic process, auxin signalling  AGL8, flower development, fruit development,  RINGLET 2, vegetative to reproductive phase transition |
| PCO4 (field)  Overlaps QTL fruit set (polytunnel)  Close to fruit set (field) | LG 3 76cM | 4615 (27543)  1320 (38675)  922 (83608)  1082:Ri9022* (44162)  1082:Ri9022*  43:Ri9022* (302968) | AT1G79730  AT1G71930  AT1G79750  AT3G13960  AT1G72570  AT1G72570 | EFL7, negative regulation of flowering by elevating FLC  NAC-17, cell wall organisation, xylem formation, response to auxin & brassinosteroid  NADP-Malic enzyme, embryo development ending in seed dormancy  GRF5, flower development    Ethylene signalling, development  Integrase, ethylene activated signalling, multicellular organism development |
| PCO4 (field) | LG 5 17cM | 1754 (29208)  3436 (18272)  715 (105361)  3442:Rub35a* (21648)  398 (66776)  398 | AT3G54720  AT5G48670  AT1G27600  AT5G24860  AT5G48600  AT3G18990 | AMP1, embryo development, flower development, meristem development, negative regulation flower development  AGL80, cell development and function  I9H, Cell wall organisation  ATFPF1, positive regulation of flower development, GA response  ATCAP-3, embryo development ending in seed dormancy  VRN1, regulation of flower development, repression FLC in vernalised plants |
| PCO4 (field)  Overlaps QTL green/red (field) and PCO2 (field), adjacent to set (field)& green (field) | LG 6  5cM | 4040 (15894)  6600 (14323)  534 (57152) | AT3G54180  AT2G34980  AT5G16715 | CDC2-LIKE, mitotic cell cycle control  SETH1, Pollen germination, pollen tube growth  EMB2247, embryo development ending in seed dormancy |

*Scaffolds positioned due to location of previously mapped markers
