# Supplementary material for: Enhancement of Glen Moy x Latham raspberry linkage map using GbS to further understand control of developmental processes leading to fruit ripening
Source: BMC Genet. 2018 Aug 15;19:59. doi: 10.1186/s12863-018-0666-z (PMC6094467; doi:10.1186/s12863-018-0666-z)
Supplement: Supplementary file 5 — Table S2. Two non-related regions with associated scaffolds within 2 cM and any potential developmentally/ripening related genes. This table examines regions not identified in ripening to ensure the ripening related genes are not chance associations. (DOCX 17 kb) [file 12863_2018_666_MOESM5_ESM.docx]

Table S2. Two non-related regions with associated scaffolds within 2cM and any potential developmentally/ripening related genes.

|  | Linkage group | Scaffolds | TAIR | Potential function in development |
| --- | --- | --- | --- | --- |
| Close to QTL for root rot resistance and root growth  [23] | 6  73cM | \| 27 (513069) \| \| --- \| \| 49 (361216) \| \| 41 (313300) \| \| 252 (86179) \| \| 49 (361216) \| \| 23 (418895) \| \| 822 (44584) \| \| 109 (149845) \| \| 46 (292920) \| \| 18 (594048) \| \| 7 (604353) \| \| 63 (251332) \| \| 231 (90145) \| \| 121 (146762) \| \| 70 (203438) \| \| 55 (365376) \| \| 62 (226968) \| \| 46 (292920) \| \| 28 (392062) \| \| 109 (149845) \| \| 70 (203438) \| \| 55 (365376) \| | None  None  None  None  None  None  None  None  None  AT4G35550  None  None  None  None  None  None  None  None  None  None  None  None  None  None | WOX-13 possible role in multicellular organism development but expressed in lateral root primordium thus role more likely in root production |
| No related QTL identified to date | LG 4  21cM | \| 9668 (10141) \| \| --- \| \| 108 (150386) \| \| 4759 (13706) \| \| 2177 (25528) \| \| 2684 (46562) \| \| 9668 (10141) \| \| 3595 (17609) \| \| 1153 (54948) \| \| 7991 (7296) \| \| 7550 (7924) \| \| 151 (128675) \| \| 499 (89619) \| \| 1325 (34494) \| \| 203 (98406) \| \| 7600 (18152) \| \| 3406 (29293) \| \| 14844 (2998) \| | None  None  None  None  None  None  None  None  None  None  None  None  None  None  None  None  None  None  None |  |
